# One minute ultrasound-assisted gold and platinum-decorated ZnO photocatalyst for ibuprofen degradation

**DOI:** 10.1039/d6na00485g

**Published:** 2026-09-02

**Authors:** Gabriele Manca, Valentín García-Caballero, Ludovico Valli, Juan José Giner-Casares, Manuel Cano, Simona Bettini, Gabriele Giancane

**Affiliations:** a Dipartimento di Scienze e Tecnologie Biologiche e Ambientali, Università del Salento Via Monteroni Lecce I-73100 Italy simona.bettini@unisalento.it; b Department of Chemistry, Biology and Biotechnology, Centro di Eccellenza per i Materiali Innovativi Nanostrutturati (CEMIN), University of Perugia via Elce di Sotto 8 Perugia 06123 Italy; c Departamento de Química Física y T. Aplicada, Instituto Químico para la Energía y el Medio Ambiente (IQUEMA), Facultad de Ciencias, Campus de Rabanales, Universidad de Córdoba Córdoba Spain q82calum@uco.es; d Consorzio Interuniversitario Nazionale per la Scienza e la Tecnologia dei Materiali, INSTM Via G. Giusti Firenze I-50121 Italy; e Department of Cultural Heritage, University of Salento Via D. Birago 64 Lecce 73100 Italy

## Abstract

The occurrence of ibuprofen in surface and wastewater is an emerging environmental concern due to its incomplete removal in conventional water treatment systems. In this work, ZnO-based hybrid photocatalysts decorated with noble metal nanoparticles (ZnO@Au and ZnO@Pt) were prepared through an ultrasound-assisted functionalization of preformed ZnO microstructures and evaluated for the photocatalytic degradation of ibuprofen in water. Structural, morphological, and optical characterization confirmed that the noble metal decoration preserved the wurtzite ZnO structure and the flower-like nanosheet morphology, while producing negligible changes in the optical band gap. Noble metal decoration significantly enhanced the photocatalytic activity of ZnO, with ZnO@Pt exhibiting the highest degradation efficiency under UV irradiation. After 90 min, ZnO@Pt achieved 90.2% ibuprofen degradation, compared with 36.1% and 8.9% for ZnO@Au and pristine ZnO, respectively. In addition, ZnO@Pt showed an apparent pseudo-first-order rate constant on the order of 10^−2^ min^−1^, approximately one and two orders of magnitude higher than those of ZnO@Au and bare ZnO, respectively. The improved photocatalytic performance may be related to a more efficient charge separation promoted by Pt nanoparticles. ZnO@Pt also retained significant activity in a more complex mineral water matrix despite the presence of competing inorganic species and natural organic matter. Overall, the results suggest that ultrasound-assisted noble metal decoration is a simple and environmentally friendly strategy to improve the photocatalytic performance of ZnO-based materials for water treatment applications involving pharmaceutical pollutants.

## Abstract 2

The occurrence of ibuprofen in surface and wastewater is an emerging environmental concern due to its incomplete removal in conventional water treatment systems (image created with the assistance of FigureLabs).

## Graphical abstract

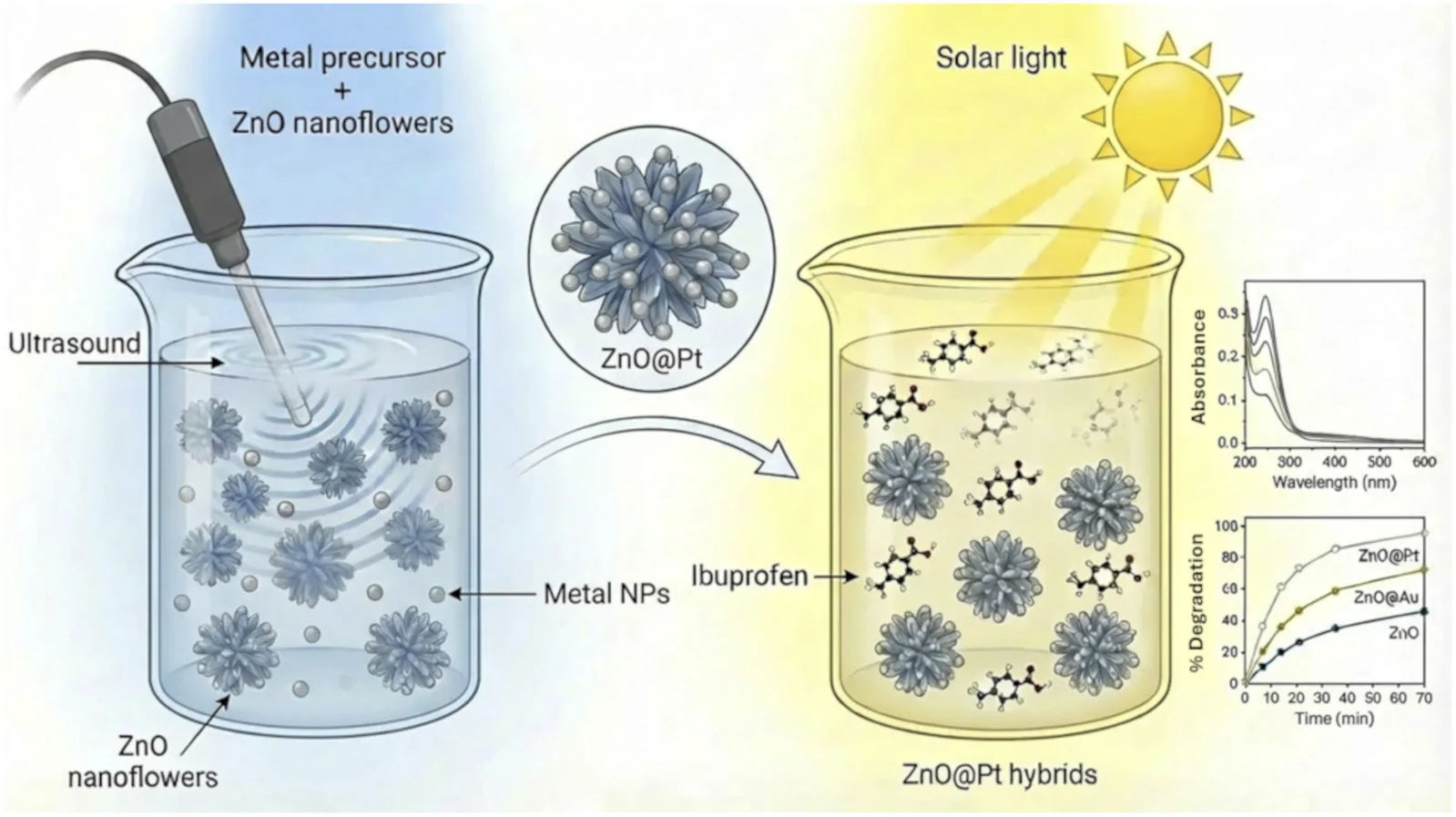

## 1 Introduction

Emerging contaminants in aquatic environments, such as pharmaceuticals, hormones and pesticides, are increasingly recognized as a serious challenge for water resource management worldwide and in Europe in particular.^1^

These compounds are detected in rivers, lakes, groundwater and treated wastewater effluents, mainly because conventional wastewater treatment plants are not designed to remove persistent, bioactive organic micropollutants.^2–7^ Among them, pharmaceuticals are of particular concern since they are continuously discharged from domestic and hospital use, are only partially metabolized, and can reach aquatic ecosystems *via* insufficiently treated effluents.^3^ In this scenario, ibuprofen (IBU) is one of the most widely consumed non-steroidal anti-inflammatory drugs (NSAIDs) and is now regarded as a representative pharmaceutical emerging contaminant.^8–11^ From a physicochemical viewpoint, IBU is a weak acid (p*K*_a_ ≈ 4.9), predominantly present in its anionic form at circumneutral pH. Among the different techniques proposed for IBU removal, such as adsorption, advanced biological treatment, ozonation, membrane processes and electrochemical methods,^10,12–14^ Advanced Oxidation Processes (AOPs) have attracted particular interest because they rely on the *in situ* generation of highly reactive oxygen species capable of non-selectively oxidizing organic pollutants.^4,15^ Within AOPs, heterogeneous photocatalysis based on wide-bandgap semiconductors such as TiO_2_ and ZnO is a widely studied strategy, owing to its ability to generate strongly oxidizing radicals upon band-gap excitation in the presence of oxygen and water.^16,17^ Since the pioneering work on photoelectrochemical water splitting by Fujishima and Honda, semiconductor photocatalysts have been intensively investigated for environmental remediation.^17,18^ Under irradiation, the formation of reactive oxygen species (ROS) is promoted, enabling the chemical degradation of IBU *via* decarboxylation, aromatic hydroxylation, and subsequent ring opening, leading ultimately to mineralization or to smaller oxygenated by-products.^19^ ZnO-based photocatalysts have been widely explored for the degradation of pharmaceuticals and dyes due to their suitable band gap, relatively high electron mobility and ease of synthesis in a variety of nanostructured morphologies.^11^

However, pristine ZnO often suffers from fast electron–hole recombination under light irradiation, which can strongly reduce its photocatalytic performance.^16,17^ In IBU removal, commercial or lab-made ZnO systems can achieve high degradation under UV light, but optimal performance typically requires relatively high catalyst dosages, careful pH adjustment or the use of additives such as H_2_O_2_ or sacrificial agents to boost radical generation and suppress charge recombination.^11,16,17^ Strategies to improve ZnO performance include coupling with other oxides or carbonaceous materials and, importantly, decorating ZnO with noble metals such as Au or Ag to act as electron sinks and interfacial catalytic sites, enhancing charge separation and interfacial redox processes in aqueous media.^1,11^ Recent studies have therefore focused on engineered metal–ZnO interfaces and hybrid architectures, where noble metals deposited on ZnO modulate band bending, act as charge-separation centers and provide additional surface sites for interfacial reactions.^11^ Alongside these developments, a variety of ZnO- and metal oxide-based photocatalysts have also been investigated, including carbon-doped ZnO, graphene–TiO_2_ nanocomposites, red phosphorus/ZnO nanohybrids, NiO–rGO hybrids, WO_3_ nanoparticles and related systems.^20–27^

In this context, we reported an ultra-rapid piezo-assisted route to fabricate ZnO-based metal/semiconductor hybrid interfaces by simple sonication. In particular, the piezoelectric behavior of ZnO under ultrasonic mechanical stimulation drives interfacial redox reactions that promote the *in situ* reduction of metal ions directly onto the zinc oxide.^28^

ZnO- and TiO_2_/ZnO-based photocatalysts have been explored for ibuprofen degradation in water, often achieving substantial removal but typically requiring relatively high catalyst dosages, specific UV sources, pH adjustment or the addition of oxidants and sacrificial agents to sustain radical generation and mitigate charge recombination.^11,16,17,19^

Noble-metal-modified ZnO systems have also been reported for the degradation of pharmaceuticals and related contaminants. For instance, Au- and Pt-decorated (002)-oriented ZnO hollow spheres were reported to efficiently degrade ibuprofen under UV irradiation. However, these materials were prepared through a multi-step synthetic route involving carbohydrate-derived carbon templates, high-temperature calcination and subsequent deposition of 1 wt% noble-metal.^29^

More generally, Au-, Ag- and Pt-modified ZnO photocatalysts are often prepared by sol–gel, photoreduction, flame-based or other multi-step wet-chemical routes, which provide efficient metal/semiconductor interfaces but generally require more complex synthetic protocols and, in many cases, relatively high noble-metal loadings.^30–33^ Nevertheless, the high cost and limited abundance of noble metals such as Au and, particularly, Pt remain important drawbacks for their large-scale application. Therefore, synthetic strategies capable of exploiting their outstanding catalytic properties while minimizing the required metal loading are particularly desirable, as they may mitigate the overall economic impact associated with their use.

Besides, ultrasound-assisted synthesis has emerged as a versatile strategy for preparing ZnO-based photocatalysts and hybrid nanostructures under mild conditions, yet its use for the ultra-rapid construction of noble-metal/ZnO interfaces specifically aimed at pharmaceutical degradation remains comparatively underexplored.^29,31,34–39^ By means of this approach, three representative materials, pristine ZnO, ZnO@Au as a model plasmonic metal/ZnO interface, and ZnO@Pt as a high-work-function Schottky-type metal/ZnO interface, have been selected for photocatalytic IBU degradation under UV/solar-simulated irradiation.

## 2 Experimental section

### 2.1 Materials

All chemicals were of analytical grade. Zinc sulphate heptahydrate (ZnSO_4_·7H_2_O, 99.95%, Merck), IBU (≥98% Sigma-Aldrich), terephthalic acid (TA, ≥98%, Thermo Fisher Scientific), nitro blue tetrazolium chloride (NBT, ≥98%, Thermo Fisher Scientific), silver nitrate (AgNO_3_, ≥99%, Alfa Aesar) and triethanolamine (TEA, ≥99,8%, Chem-Lab Analytical) were used without further purification. All aqueous solutions were prepared using Milli-Q water (18.2 MΩ cm).

### 2.2 Synthesis of ZnO@M hybrid materials

Flower-like ZnO microstructures were prepared according to a previously reported procedure and, in brief, were synthesized by chemical precipitation using zinc sulphate heptahydrate (ZnSO_4_·7H_2_O, 99.95%, Merck) as the Zn precursor and NaOH, followed by ageing at 90 °C, washing and drying.^40^ Then, metal-decorated hybrids (ZnO@M, M = Au, Pt) were synthesized *via* an ultrasound-assisted piezo-driven protocol.^28^

Platinum(ii) chloride (PtCl_2_, 74%, Fluka Chemika) and chloroauric acid trihydrate (HAuCl_4_·3H_2_O, ≥99%, Merck) were used as Pt and Au precursors, respectively.

In a typical synthesis, pristine ZnO was used at a concentration of 1 mg mL^−1^ in a Milli-Q water/ethanol mixture (1.5 : 1 v/v), and the corresponding metal salt was added to reach a final concentration of 10^−4^ M, then 40 µL aliquots of 5 × 10^−3^ M stock solutions of HAuCl_4_·3H_2_O and PtCl_2_ were added to the ZnO suspension, and the final reaction volume was adjusted to 2 mL. The resulting suspension was directly subjected to ultrasonic irradiation for 1 min in a conventional ultrasonic bath (35 kHz, 100 W nominal power), operated at room temperature and in the dark. During sonication, the reaction vessel was placed in the central region of the bath and the bath temperature was monitored, remaining close to ambient throughout the treatment. After sonication, the powders were collected by centrifugation, thoroughly washed with Milli-Q water and ethanol to remove residual ions, and finally dried at room temperature before further characterization and photocatalytic tests. As discussed in our previous study on piezo-triggered ZnO@Au interfaces,^28^ the implosion of cavitation bubbles in contact with piezoelectric ZnO generates transient piezopotentials and internal electric fields that can promote noble-metal ion reduction through two complementary contributions. On the one hand, the mechanical deformation of ZnO leads to the formation of piezo-charges within the semiconductor, which guide free carriers toward Au^3+^ or Pt^2+^ ions. On the other hand, the finite charge-screening length at the ZnO/liquid interface gives rise to screening charges that accumulate near the solid–liquid junction, providing local electron-rich regions in close proximity to solvated metal ions. Under the present ultrasound conditions, these piezo-charges and screening charges are expected to act together, driving the *in situ* reduction of Au^3+^ and Pt^2+^ and the rapid nucleation and growth of metal nanostructures directly on the ZnO surface. This piezo-redox mechanism is consistent with control experiments previously reported for ZnO@Au, in which noble-metal ion reduction and nanoparticle nucleation were observed only when piezoelectric ZnO was activated by ultrasound, whereas static contact, light irradiation without ultrasound and ultrasound treatment of non-piezoelectric TiO_2_ did not lead to detectable metal deposition.

### 2.3 Morphological and physicochemical characterization

Powder X-ray diffraction (PXRD) patterns were collected at room temperature on a Malvern PANalytical Aeris diffractometer operated in transmission geometry mode and equipped with a PIXcel1D solid-state detector and a Cu anode (Cu Kα radiation, *λ* = 1.5406 Å). Data were acquired over the 2*θ* range of 25–80° with a step size of 0.021°. For each sample, seven consecutive scans were recorded and averaged to improve the signal-to-noise ratio. Transmission electron microscopy (TEM) imaging and Energy Dispersive Spectroscopy (EDS) were performed using a Talos F200i microscope operating at an accelerating voltage of 80 kV. Samples were prepared by drop-casting 10 µL of the dispersed material onto 200 mesh copper grids coated with Formvar/carbon films, followed by drying under ambient conditions.

#### 2.3.1 ICP-OES analysis

Inductively coupled plasma optical emission spectroscopy (ICP-OES) analyses were performed using an iCAP 7200 ICP-OES Duo instrument (Thermo Fisher Scientific) to determine the actual noble-metal loadings of the ZnO-based hybrid materials. Prior to analysis, the powder samples were digested in aqua regia (HNO_3_/HCl = 3 : 1 v/v) for 24 h at 80 °C. The resulting solutions were then analyzed by ICP-OES to quantify the Au and Pt contents.

#### 2.3.2 Optical characterization

The optical properties of the materials were investigated by UV-vis absorption spectroscopy using an integrating sphere (PerkinElmer Lambda 650S).

The optical band gap energies (*E*_g_) were estimated from the absorbance data using the Tauc method. Assuming a direct allowed transition and considering the measured absorbance (*A*) as proportional to the absorption coefficient (*α*), Tauc plots were constructed by plotting (*Ahν*)^2^*versus* photon energy (*hν*). *E*_g_ was determined by linear fitting of the near-edge region and extrapolation of the linear portion to (*Ahν*)^2^ = 0.^41^

#### 2.3.3 Photoluminescence spectroscopy

Photoluminescence (PL) measurements were carried out using a Horiba Jobin Yvon Fluorolog spectrofluorometer in order to investigate the recombination dynamics of photogenerated charge carriers. Catalyst suspensions were prepared at a concentration of 1 mg mL^−1^ in Milli-Q water. The samples were excited at 375 nm wavelength, corresponding to the near band-edge excitation of ZnO, and the emission spectra were recorded under identical experimental conditions for all samples.

#### 2.3.4 Mott–Schottky analysis

Electrochemical Mott–Schottky measurements were performed to investigate the semiconductor properties of the materials and determine the flat-band potential by employing a Biologic VSP potentiostat/galvanostat electrochemical analyzer in a three-electrode configuration. A glassy carbon disk electrode (GCE) was used as the working electrode, a graphite rod as the counter electrode and an Ag/AgCl electrode (3 M KCl) as the reference electrode. Measurements were conducted in 0.1 M KOH (pH 13) aqueous solution. Mott–Schottky plots were acquired at three different frequencies (1, 2, and 3 kHz) over the potential range of −1.0 to +0.6 V. The working electrodes were prepared by drop-casting 20 µL of a catalyst suspension (5 mg mL^−1^) onto a GCE (5 mm diameter; Pine Instruments), followed by drying at room temperature and in the absence of light overnight. Prior to the measurements, the electrolyte solution was purged with nitrogen gas for 20 min to remove dissolved oxygen.

### 2.4 Photocatalytic experiments

#### 2.4.1 Photocatalytic IBU degradation and the reusability test

Photocatalytic degradation experiments were carried out using aqueous solutions of IBU (1 × 10^−4^ M) in Milli-Q water. The catalyst was dispersed at a concentration of 0.25 mg mL^−1^ in a total volume of 4 mL. Before illumination, the suspension was magnetically stirred in the dark for 15 min in order to establish adsorption–desorption equilibrium between the pollutant and the catalyst surface.

The photocatalytic experiments were performed using a LOT-Oriel Solar Simulator (Solar S, Class A) with a light intensity of 0.1 W cm^−2^. The light source was positioned at a distance of 10 cm from the reaction system. During irradiation, the suspension was maintained under mild magnetic stirring at room temperature.

The experimental setup for the dark adsorption–desorption equilibrium step and the subsequent IBU photodegradation experiment, as described above, is shown in Fig. S1. At regular intervals (every 10 min of effective irradiation), light exposure of the reaction suspension was temporarily interrupted by closing a shutter positioned between the light source and the reaction vessel, while the lamp remained switched on. A 1 mL aliquot of the reaction suspension was withdrawn, the catalyst particles were separated by centrifugation, and the clear supernatant was analyzed using a Cary 5000 UV-vis spectrophotometer (Agilent Technologies) by monitoring the characteristic absorption band of IBU at approximately 225 nm. After analysis, the catalyst was recovered and reintroduced into the reaction vessel, so that the catalyst loading remained essentially constant over the entire irradiation period. The photocatalytic degradation efficiency (*E*%) was calculated from the variation of the absorbance according to:*E* (%) = [(*A*_0_ − *A*_*t*_)/*A*_0_] × 100where *A*_0_ and *A*_*t*_ represent the absorbance values at the initial time and after irradiation time *t*, respectively.

Furthermore, cut-off filter experiments were carried out in order to investigate the individual role of the semiconductor and the metal nanoparticles (NPs) in the photocatalytic process by employing an optical filter with a cut-off wavelength of 399 nm.

The stability and reusability of the photocatalysts were investigated through four consecutive photocatalytic cycles.

After each photocatalytic experiment (60 min irradiation), the suspension was centrifuged to collect the solid catalyst, the supernatant was discarded, and the recovered solid was washed twice with Milli-Q water and once with ethanol to remove residual ibuprofen and intermediates. The solid was then dried at room temperature (overnight) and directly reused under the same photocatalytic conditions without any additional thermal treatment. The catalyst recovery, calculated as the mass of dried solid after each cycle relative to the initial mass, was typically in the 90–95% range, indicating that only a minor fraction of the ZnO-based material is lost during handling and separation.

#### 2.4.2 Photocatalytic ROS production and scavenging tests

Reactive oxygen species (ROS) generation experiments were carried out under the same irradiation conditions described above for the photocatalytic degradation tests. In all ROS detection experiments, the catalyst concentration was maintained at 0.66 mg mL^−1^. For each experiment, the analytical signals were collected before illumination (*T*_0_) and after 60 min of irradiation.

The formation of hydroxyl radicals (˙OH) was investigated using terephthalic acid (TA) as a fluorescent probe^42,43^ dissolved in aqueous solution at a concentration of 2.6 × 10^−3^ M. TA reacts with hydroxyl radicals to produce 2-hydroxyterephthalic acid (2-HTA) according to the following reaction:TA + ˙OH → 2-HTA

The hydroxylated product (2-HTA) is stable and highly fluorescent, whereas TA itself exhibits negligible fluorescence.

Fluorescence spectra of 2-hydroxyterephthalic acid were recorded using an excitation wavelength of 315 nm and the emission band centered at approximately 425 nm was monitored.^44–46^

The formation of superoxide radicals (˙O_2_^−^) was investigated using nitro blue tetrazolium (NBT) as a chemical probe,^47–49^ dissolved in aqueous solution at a concentration of 3 × 10^−5^ M. Upon reaction with superoxide radicals, NBT is reduced to formazan, an insoluble compound characterized by a blue–violet coloration. The reaction can be summarized as:NBT + 4˙O_2_^−^ + 2H^+^ → Formazan

The reaction was monitored using a Cary 5000 UV-vis spectrophotometer, following the evolution of the characteristic NBT absorption band at about 250 nm.

Radical scavenger experiments were carried out on pristine ZnO and on the ZnO@Pt sample. Pristine ZnO was used as the reference photocatalyst, whereas ZnO@Pt was chosen for additional mechanistic studies as a representative of the most active metal-decorated ZnO hybrids identified in this work.

Triethanolamine (TEA, 1 mM) was employed as a hole scavenger,^50–52^ whereas silver nitrate (AgNO_3_, 1 mM) was used as an electron scavenger.^53^ For experiments in the presence of TEA, IBU degradation was monitored by UV-vis absorption as described for the standard photocatalytic tests^19^ and the degradation efficiencies were evaluated after 30 and 60 min of irradiation.

The photocatalytic response was followed by monitoring the intrinsic fluorescence of IBU (excitation wavelength at 263 nm; emission band centered at approximately 288 nm), and the integrated emission areas recorded with and without AgNO_3_ were compared for each photocatalyst to evaluate the influence of electron trapping on the photodegradation process. Furthermore, photocatalytic experiments were carried out in spiked commercial mineral water (chemical physical characteristics are reported in Table S1), used as purchased. IBU was spiked to 1 × 10^−4^ M, and the catalyst loading was fixed at 0.25 mg mL^−1^, identical to the experiments in Milli-Q water. After 15 min of dark equilibration under mild magnetic stirring, the suspension was irradiated under simulated solar light, and the IBU concentration was monitored by following the intensity of the absorption band at 225 nm at three irradiation times (30, 60 and 90 min, respectively).

## 3 Results and discussion

### 3.1 Structural, morphological, optical and electronic characterization

TEM and high-angle annular dark-field (HAADF-STEM) images show that pristine ZnO consists of flower-like aggregates of nanosheets with well-defined edges (Fig. 1A and S2). This particle size is ideal to allow easy recovery after photodegradation of IBU by centrifugation. In the metal-decorated samples, HAADF-STEM images (Fig. S3 and S4) confirm the presence of the deposited metals. In ZnO@Au, Au appears as about 10 nanometer-sized dark spherical NPs (Fig. 1B), whereas in ZnO@Pt, Pt is observed as uniformly distributed bright circular features of approximately 2.5 nanometers (Fig. 1C).

**Fig. 1:**
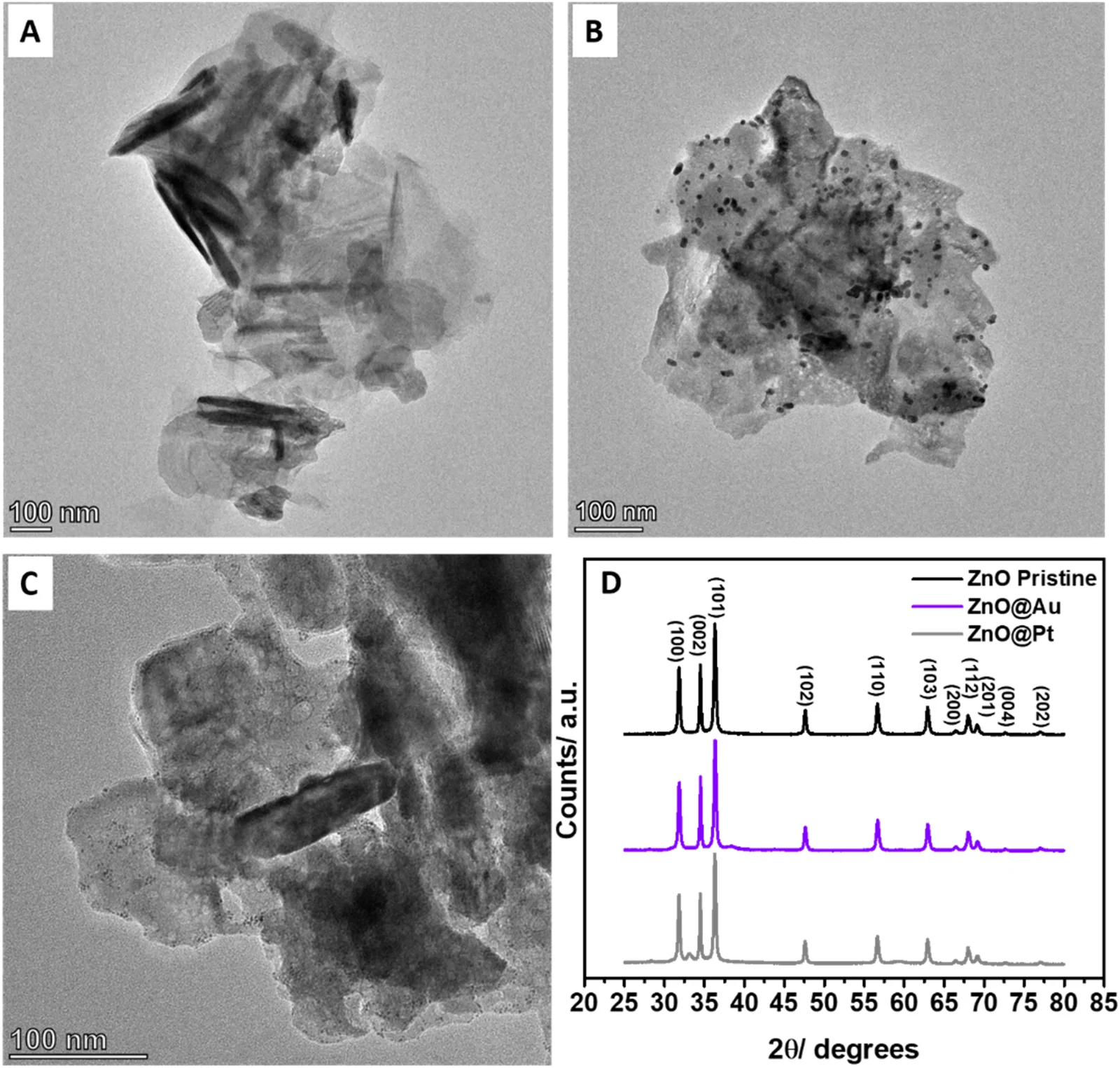
(A) TEM images of pristine ZnO with their peculiar flower-like nanosheet aggregates; (B and C) TEM images of ZnO@Au and ZnO@Pt; (D) PXRD patterns of ZnO, ZnO@Au and ZnO@Pt indexed to hexagonal wurtzite ZnO.

Particle size distributions obtained from the statistical analysis of 100 nanoparticles per sample are reported in Fig. S5, further confirming the narrow dispersion of Au and Pt sizes around these average values.

PXRD patterns of ZnO and metal-decorated samples (ZnO@Au and ZnO@Pt) (Fig. 1D) can be indexed to the hexagonal wurtzite structure of ZnO (JCPDS 36-1451), with very similar peak positions and line shapes for all materials, indicating that the piezo-assisted treatment does not introduce detectable changes in the crystal structure. A very weak additional reflection at around 2*θ* ≈ 38.3° is observed for ZnO@Au, consistent with the (111) plane of metallic gold and qualitatively supporting the presence of Au NPs, whereas no distinct Pt-related peaks can be resolved within the sensitivity of the measurement.^54^

TEM-EDS elemental mapping (Fig. S3D and S4D) highlights the presence of Au and Pt NPs homogeneously distributed over the ZnO microstructures, without evidence of the presence of large metal-rich regions at the investigated length scale. These observations indicate that the ultrasound-assisted route leads to ZnO-supported noble-metal NPs that are reasonably well dispersed on the oxide surface. The successful decoration of ZnO with noble metals was further verified by ICP-OES, which provides a quantitative assessment of the actual metal content achieved during the ultrasound-assisted synthesis. The Au- and Pt-decorated samples exhibit metal loadings of 1.46 wt% and 0.81 wt%, respectively. These values confirm that the present ultrasound-driven route yields low-wt% noble-metal/ZnO hybrid interfaces, consistent with the absence of intense metallic reflections in PXRD and with the surface-sensitive contrast observed by TEM/EDS.

Diffuse reflectance UV-vis spectroscopy (Fig. 2A) was employed to investigate the optical properties of the materials. Pristine ZnO exhibits the typical spectral profile in the near-UV region associated with the band-to-band transition of the semiconductor.^55,56^ In the visible range, both ZnO@Au and ZnO@Pt exhibit increased absorbance signal intensity compared to pristine ZnO, indicating that the presence of metal NPs enhances light harvesting beyond the intrinsic band-edge of ZnO. However, only ZnO@Au displays an additional band in the visible region, which can be attributed to the localized surface plasmon resonance (LSPR) of the Au NPs. This plasmonic band is consistent with the presence of Au NPs with sizes in the range of 5–10 nm, as observed by TEM.^57,58^ In contrast, ZnO@Pt displays a global increase in absorbance values, in line with the non-plasmonic character of Pt in this spectral window and suggesting that Pt-induced visible-light absorption arises from interfacial electronic effects and defect-related states rather than from a LSPR band.

**Fig. 2:**
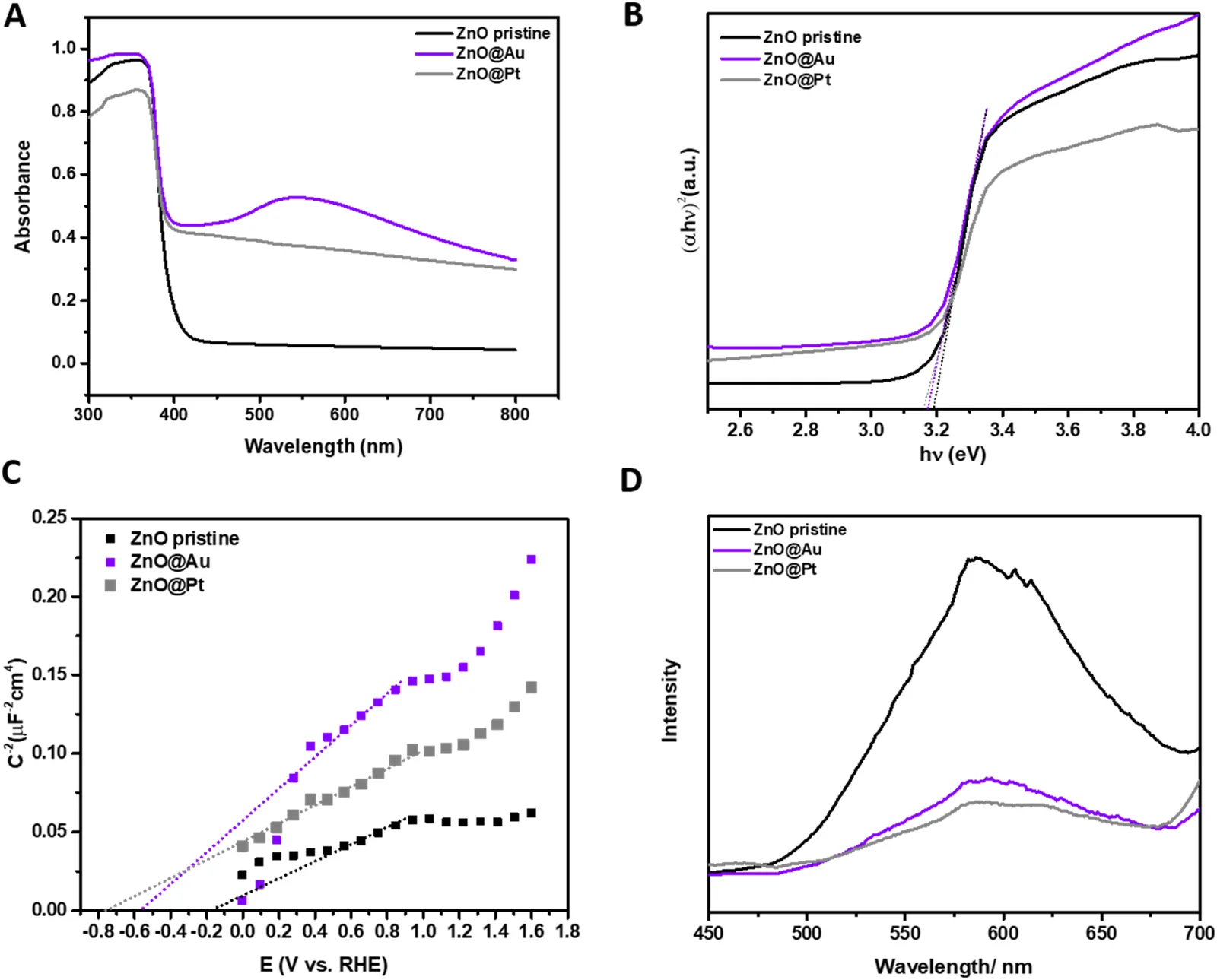
(A) Diffuse reflectance UV-vis spectra of pristine ZnO, ZnO@Au and ZnO@Pt; (B) Tauc plots estimating the optical band gaps; (C) Mott–Schottky plots at 2 kHz in 0.1 M KOH confirming the n-type character and flat-band potentials of the samples; (D) photoluminescence spectra (*λ*_exc_ = 375 nm) of ZnO, ZnO@Au and ZnO@Pt.

The optical band gap values were estimated from the absorbance spectra using the Tauc method (Fig. 2B). The calculated band gap energies were found to be 3.19, 3.17 and 3.16 eV for pristine ZnO, ZnO@Au and ZnO@Pt, respectively, indicating only negligible variations among the investigated materials and suggesting that the intrinsic electronic structure of ZnO remains essentially unchanged after metal decoration.

The obtained results indicate that the introduction of Au and Pt NPs does not significantly modify the bulk structural and optical properties of ZnO, while the noble metal species are mainly located on the surface of the semiconductor. This configuration is particularly advantageous for photocatalytic applications, as surface metal NPs can act as charge extraction sites, while preserving the intrinsic electronic structure of the semiconductor.

Although the optical band gap values remain essentially unchanged after metal decoration, the electronic properties of the investigated materials exhibit significant variations, as revealed by Mott–Schottky analysis (Fig. 2C and S6). The Mott–Schottky plots recorded for all samples display positive slopes,^30,59^ confirming the typical n-type semiconductor behavior of ZnO. From the linear regions of the Mott–Schottky plots, the flat band potentials (*E*_fb_) were determined by extrapolating the straight-line fits to their intersection with the potential axis. The measured potentials, initially obtained with respect to the Ag/AgCl reference electrode, were subsequently converted *vs.* RHE (eqn (1))^60,61^ and finally to the vacuum energy scale using the following relationship (eqn (2)):^62^

1 *E* (RHE) = *E* (Ag/AgCl) + *E*^0^ (Ag/AgCl) + 0.0591 pH

With *E*^0^ (Ag/AgCl) = 0.205 V

2 *E*_vac_ (eV) = −(*E*_vs RHE_ (V) + 4.44)

The resulting flat band potentials were calculated to be −4.22, −3.77 and −3.65 eV for pristine ZnO, ZnO@Au and ZnO@Pt, respectively.

The flat-band potential becomes progressively more positive going from ZnO to ZnO@Au and ZnO@Pt, with the largest shift observed for ZnO@Pt, consistent with stronger band bending and a larger built-in electric field at the Pt/ZnO interface supporting the formation of a more effective Schottky contact in ZnO@Pt than in ZnO@Au.

For n-type semiconductors, flat band potential can be reasonably approximated to the position of the conduction band (CB) edge. Therefore, these values were used to estimate the conduction band energies of the investigated materials. The valence band (VB) positions were subsequently calculated by subtracting the optical band gap values obtained from the Tauc analysis of the absorbance data from the corresponding CB energies. This procedure allowed the determination of the complete band structure of the investigated systems, enabling a direct comparison of the band energetics of pristine ZnO and the metal-decorated materials. The obtained donor density values (*N*_d_) are in the order of 10^20^ cm^−3^ for all investigated samples. These comparable values further indicate that the introduction of noble metal NPs does not significantly modify the bulk carrier concentration of ZnO. On the other hand, the clear shift of flat band potential is observed upon metal decoration. Such a shift can be attributed to the modification of the interfacial electronic structure rather than to a change in the bulk electronic properties of ZnO. The band structure parameters of the investigated materials are summarized in Table S2.

Specifically, the conduction band edge shifts progressively from −4.22 eV for ZnO to −3.85 and −3.65 eV for ZnO@Au and ZnO@Pt, respectively. Such a trend indicates a progressively stronger perturbation of the electronic structure at the metal–semiconductor interface,^63,64^ with the largest effect observed for the Pt-decorated material.

This behavior can be rationalized considering the work functions of the synthesized materials. When a metal with a work function higher than ZnO is deposited onto its surface, Fermi level alignment induces electron transfer from the semiconductor toward the metal until electrostatic equilibrium is reached. This process leads to the formation of a space charge region in the semiconductor, giving rise to band bending near the metal–semiconductor interface.^64,65^ The progressively larger shift observed for ZnO@Au and especially for ZnO@Pt therefore suggests the formation of increasingly strong interfacial electric fields, consistent with the higher work function of Pt compared with Au.^66^ To clarify how the metal decoration affects the behavior of photogenerated charge carriers, we performed photoluminescence (PL) measurements (Fig. 2D), using excitation wavelengths close to the near band edge of ZnO (375 nm). In semiconductor materials, PL emission mainly arises from the radiative recombination of photogenerated electron–hole pairs. Compared with pristine ZnO, both the hybrid materials exhibit a markedly reduced PL intensity, showing that a significant portion of the fate of photogenerated carriers is influenced by the presence of metal decoration, reducing the radiative recombination.

Compared with ZnO and ZnO@Au, ZnO@Pt shows the largest flat-band shift and the strongest PL quenching, pointing to more pronounced band bending at the Pt/ZnO interface and more efficient suppression of radiative electron–hole recombination.

This behavior is consistent with efficient interfacial charge transfer between ZnO and the metal NPs, which promotes a more effective spatial separation of photogenerated electrons and holes. To better visualize these trends, a schematic band diagram referenced to the RHE scale was constructed for ZnO, ZnO@Au and ZnO@Pt (Scheme 1).

**Scheme 1:**
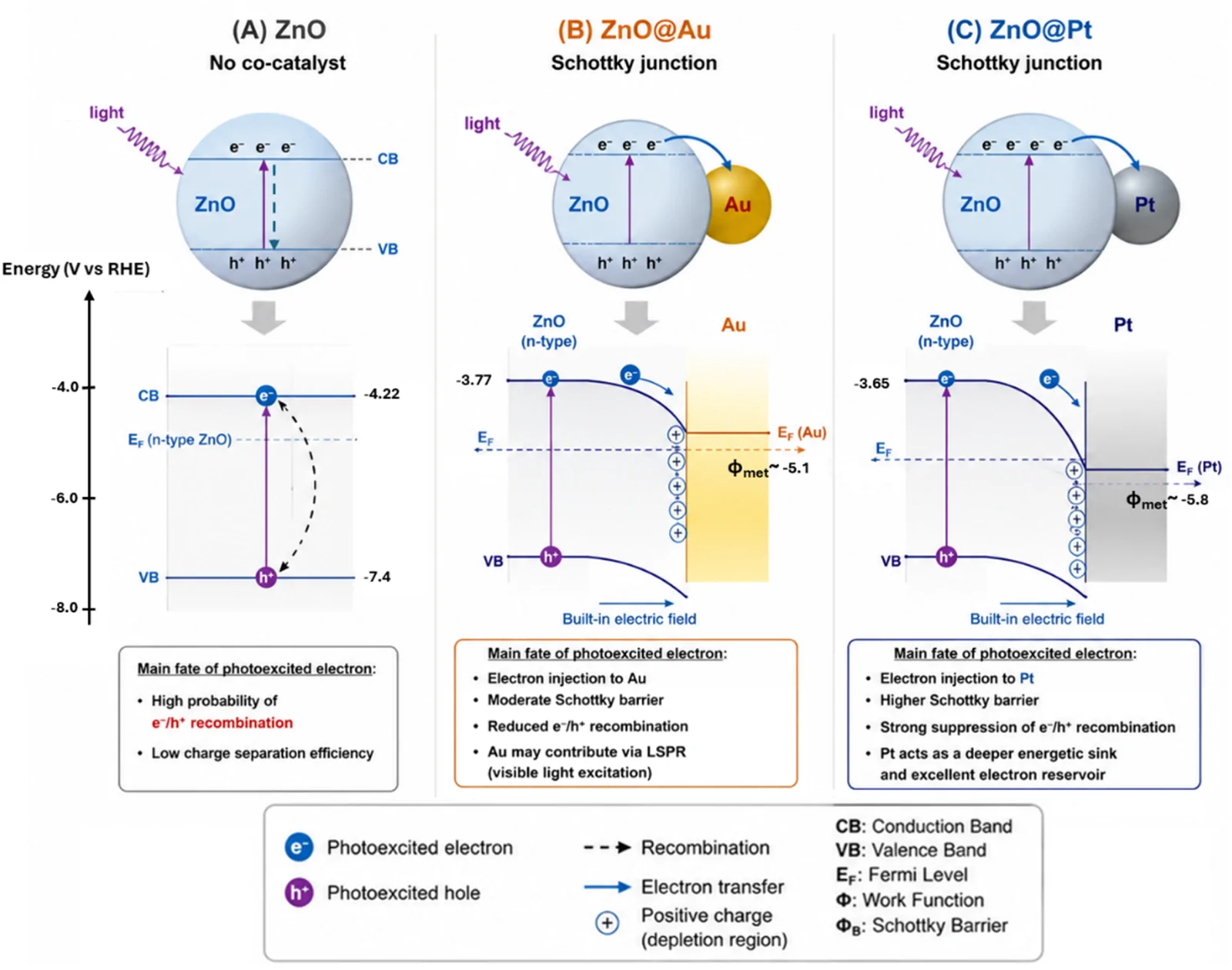
Schematic band diagrams of (A) pristine ZnO, (B) ZnO@Au and (C) ZnO@Pt referenced to the reversible hydrogen electrode (RHE). The schemes illustrate the alignment of the conduction band (CB), valence band (VB), Fermi level (*E*_f_) and metal work functions, as well as the formation of Schottky junctions and built-in electric fields at the ZnO/Au and ZnO/Pt interfaces. This scheme was created with the assistance of FigureLabs.

### 3.2 Photocatalytic degradation of IBU

The photocatalytic activity of the synthesized materials was evaluated through the degradation of IBU under simulated solar irradiation. Prior to illumination, the catalyst suspensions were magnetically stirred in the dark for 15 min to establish adsorption–desorption equilibrium between the catalyst surface and the pollutant molecules (Fig. S7A–C). The IBU absorption band is located at about 225 nm, and it was monitored under dark conditions for 30 min at 5 min intervals. A plateau was reached after 10 min; therefore, 15 min was selected as the equilibrium time for adsorption–desorption.

The variations of IBU concentration during irradiation were monitored by UV-vis spectroscopy by following its characteristic absorption band. The degradation profiles obtained for the investigated catalysts are shown in Fig. 3A–C.

**Fig. 3:**
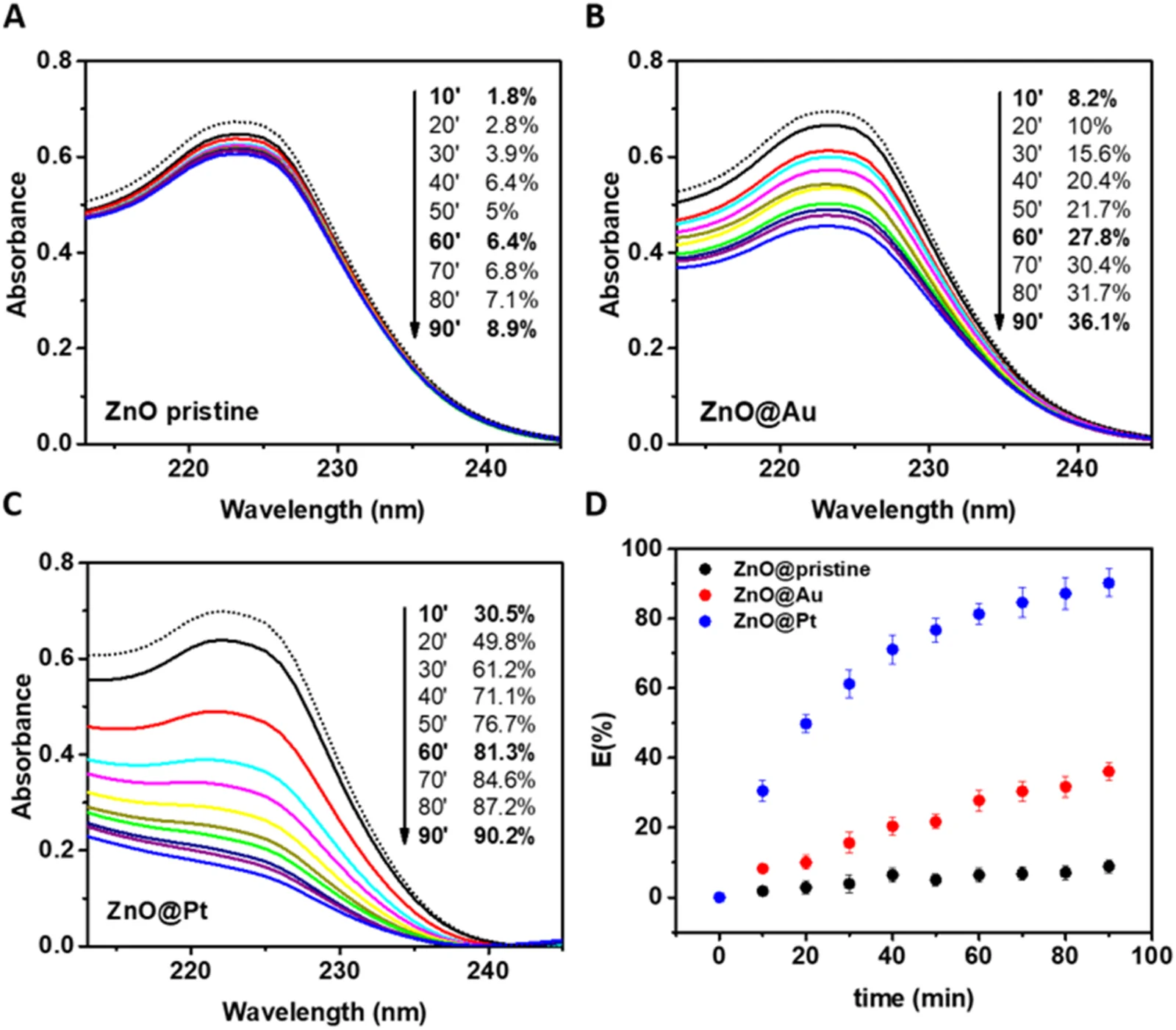
Photocatalytic degradation of IBU under simulated solar irradiation: (A–C) evolution of the UV-vis absorption spectra of IBU solution in the presence of ZnO, ZnO@Au, and ZnO@Pt catalysts, respectively, recorded at different irradiation times. In each panel, the dotted black curve corresponds to the 0 min spectrum of IBU at its initial concentration (1 × 10^−4^ M) while the progressive decrease in the characteristic absorption band of IBU at approximately 225 nm indicates the gradual degradation of the pollutant. (D) Corresponding degradation efficiencies *E* (%) as a function of irradiation time for the investigated photocatalysts.

Under the investigated conditions, pristine ZnO exhibited only a limited photocatalytic activity, reaching a degradation efficiency of 8.9% after 90 min of irradiation. In contrast, the introduction of noble metal NPs significantly improved the photocatalytic performance of the ZnO-based systems. In particular, ZnO@Au showed a moderate enhancement, reaching a degradation efficiency of 36.1% after 90 min, whereas ZnO@Pt exhibited a dramatic increase in photocatalytic activity, achieving more than 90% degradation within the same irradiation time.

A photolysis control experiment was also performed under the same operational conditions but in the absence of any catalyst. The resulting ibuprofen degradation was very limited (≈2.2% after 90 min, see Fig. S7D), confirming that direct photolysis is negligible under the conditions used in this work.

This result clearly demonstrates the strong catalytic effect induced by Pt NP decoration. The linear relationships obtained from the corresponding kinetic plots (Fig. 4A) confirm that the degradation process follows pseudo-first-order kinetics for all the investigated catalysts. The reaction kinetics were evaluated assuming a pseudo-first-order model according to:

3 ln(*A*_0_/*A*) = *kt*

where *k* represents the apparent rate constant.

**Fig. 4:**
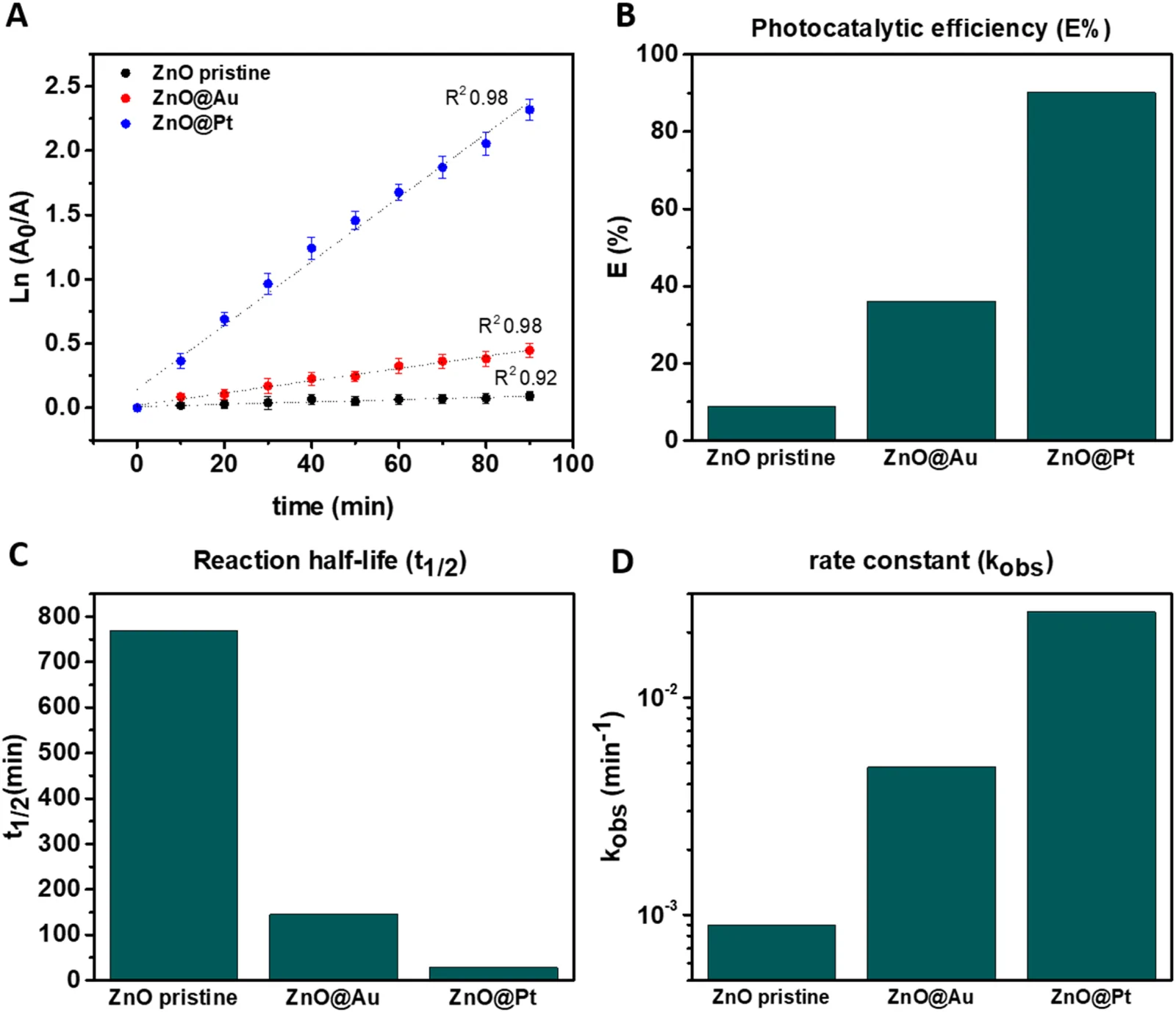
(A) Pseudo-first order fitting of pristine ZnO, ZnO@Au and ZnO@Pt catalysts. Comparison of photocatalytic performance of pristine ZnO and metal-decorated systems (Au and Pt), showing (B) enhanced efficiency and kinetics upon surface modification (C and D).

The apparent rate constants and the corresponding half-life values derived from the kinetic analysis, summarized in Table S3, highlight the strong influence of noble metal decoration on the photocatalytic activity.

Specifically, pristine ZnO exhibits a very low-rate constant of 9 × 10^−4^ min^−1^, while the introduction of Au NPs increases the reaction rate to 4.78 × 10^−3^ min^−1^, corresponding to an approximately five-fold increase compared to the pristine material. An even more remarkable enhancement is observed for ZnO@Pt, which exhibits a rate constant of 2.48 × 10^−2^ min^−1^, nearly 28 times higher than that of pristine ZnO and approximately five times higher than that of ZnO@Au. This remarkable increase clearly highlights the beneficial role of Pt NPs in accelerating the photocatalytic degradation process. Interestingly, ICP-OES shows that ZnO@Pt contains a lower metal loading (0.81 wt%) than ZnO@Au (1.46 wt%), yet it exhibits a markedly higher photocatalytic activity toward ibuprofen degradation. This finding indicates that the superior performance of ZnO@Pt cannot be simply attributed to a larger amount of deposited metal, but rather to a more effective interfacial electronic interaction between Pt and ZnO, consistent with the stronger flat-band shift and photoluminescence quenching observed for the Pt-decorated sample.

The strong catalytic enhancement is also reflected in the corresponding half-life values (*t*_1/2_), calculated according to:

4 *t*_1/2_ = ln 2/*k*

For pristine ZnO, a half-life of approximately 770 min is obtained, indicating a very slow degradation process. The introduction of Au NPs reduces this value to 145 min, while ZnO@Pt shows a drastically shortened half-life of only 28 min, confirming the faster degradation kinetics promoted by Pt decoration.

When compared with ZnO-based photocatalysts previously reported for IBU reduction, which often rely on higher catalyst loadings, specific UV lamps, pH adjustment and/or oxidant addition, ZnO@Pt displays comparable or superior degradation efficiencies and rate constants. It is worth stressing that the ZnO@Pt photocatalyst, prepared through an ultra-fast, additive-free ultrasound-assisted route, works under mild conditions, low catalyst dosage, relatively high initial IBU concentration and simulated solar irradiation.^11,16,17,19,67^

As will be discussed in the following sections, this improvement can be rationalized by considering the electronic interaction at the metal–semiconductor interface, which promotes more efficient separation of photogenerated charge carriers and facilitates interfacial electron transfer processes.

Finally, the stability and reusability of the ZnO@Pt catalyst were evaluated over five consecutive photocatalytic cycles, giving IBU removal efficiencies of 82, 78, 71, 60 and 58% in the first to fifth run, respectively (Fig. S8). The decrease in photodegradation performance upon recycling could be expected. In fact, in the absence of any regeneration steps between different cycles, deactivation phenomena such as catalyst surface poisoning/fouling and light-induced surface changes can occur.^68–71^

Although a gradual decrease was observed, the catalyst retained over 70% efficiency after three cycles and still removed nearly 60% of IBU in the fifth run, indicating good stability and practical reusability for repeated water treatment operations. To better understand the physical and chemical mechanisms that rule the IBU degradation, cut-off filter experiments were carried out on all the materials. In particular, a 399 nm cut-off filter was used in order to exclude the photons able to promote the electron excitation on ZnO. The corresponding degradation efficiencies at 30 and 60 min for ZnO, ZnO@Au and ZnO@Pt, with and without the cut-off filter, are summarized in Fig. 5 (bar chart), while the evolution of the UV-vis absorption spectra during irradiation is reported in Fig. S9.

**Fig. 5:**
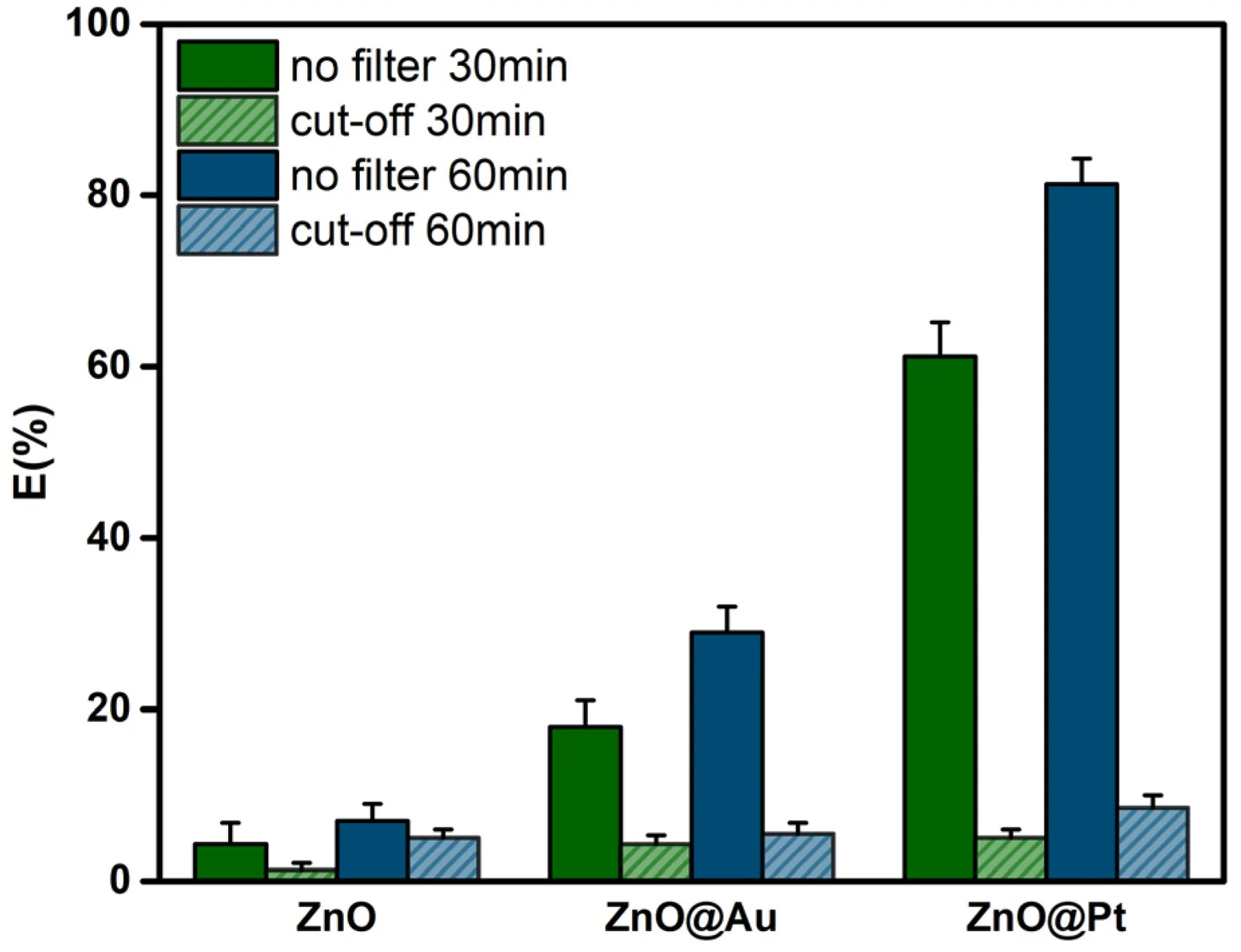
Photocatalytic degradation efficiencies *E* (%) of IBU after 30 and 60 min of irradiation for ZnO, ZnO@Au and ZnO@Pt, in the absence and in the presence of the 399 nm cut-off filter (*λ* >399 nm).

Using the 399 nm cut-off filter, a pronounced decrease in photocatalytic performance is observed for all samples: the degradation efficiencies after 30 and 60 min drop to 1.3 and 5.0% for pristine ZnO, 4.3 and 5.5% for ZnO@Au, and 5.0 and 8.5% for ZnO@Pt, respectively. In particular, for ZnO@Pt the degradation at 60 min decreases by more than one order of magnitude compared with full solar spectrum illumination, clearly indicating that efficient IBU removal cannot be sustained without ZnO band-gap excitation. For pristine ZnO, the very low efficiencies detected under cut-off conditions can be regarded as negligible within experimental uncertainty and are fully consistent with the UV-vis diffuse reflectance spectra, which show no absorption above 400 nm.

The slightly higher apparent values measured for ZnO@Au and ZnO@Pt under *λ* > 399 nm remain far below those obtained under full-spectrum irradiation and mainly reflect their residual optical absorption in the visible range, arising from the Au plasmon band and interfacial states at the Pt–ZnO junction, suggesting that any contribution of the metal NPs to genuine visible-light photocatalysis is negligible under the present conditions. These results confirm the findings already obtained from the optical and electronic characterization of the examined photocatalysts. It can therefore be inferred that the Au and Pt NPs primarily act as interfacial sinks for photogenerated electrons at the metal–ZnO junction, boosting the ZnO-driven process instead of functioning as autonomous visible-light photocatalysts.^28,72–75^ This interpretation is supported by the marked quenching of the near-band-edge photoluminescence already discussed for ZnO@Au and especially ZnO@Pt compared with pristine ZnO, which points to more efficient suppression of radiative electron–hole recombination in the metal-decorated samples, consistent with the formation of Schottky-like contacts.^65,76,77^ Under these conditions, the superior photocatalytic performance of ZnO@Pt compared with ZnO@Au and pristine ZnO can be rationalized by the combined effect of Schottky junction formation, efficient electron trapping at Pt nanoparticles and the intrinsic catalytic properties of Pt toward oxygen reduction.^78^ The higher work function of Pt relative to Au induces a stronger upward band bending in ZnO, resulting in a larger built-in electric field at the Pt/ZnO interface that promotes spatial separation of photogenerated electrons and holes and reduces their recombination probability. Electrons transferred from ZnO to Pt are efficiently trapped on the metal nanoparticles, participating in interfacial oxygen reduction, while the corresponding holes accumulated in the ZnO valence band drive oxidation processes. The markedly enhanced activity of ZnO@Pt with respect to ZnO@Au is therefore attributed to the more effective charge separation and faster interfacial redox kinetics established at the Pt/ZnO interface.

### 3.3 Scavenging tests and photocatalytic ROS production studies

The role of photogenerated charge carriers was further investigated by scavenger experiments with TEA and AgNO_3_ (Fig. S10) carried out on the best performing catalyst for IBU degradation, that is ZnO@Pt.

In the presence of the hole scavenger TEA, IBU degradation efficiency was strongly reduced, decreasing from 81.3% under standard conditions to only 7.7%. This drastic inhibition clearly indicates that valence band holes, and the oxidative species derived from them, are crucial for IBU photodegradation. In contrast, the addition of AgNO_3_, used as an electron scavenger and monitored through the fluorescence emission of IBU, does not suppress ibuprofen degradation, but rather a slight increase in the degradation efficiency is observed compared to the scavenger-free experiments. This trend can be rationalized by considering the role of Ag^+^ as an efficient electron scavenger, which further suppresses electron–hole recombination and prolongs the lifetime of photogenerated holes at the ZnO-based interfaces. In agreement with literature reports, AgNO_3_ and other electron acceptors, indeed, act as sacrificial electron sinks and, in some systems, even improve the photocatalytic degradation of organic dyes and pollutants.^79,80^

In order to further clarify this crucial point, ROS production studies were performed. Hydroxyl radical formation was investigated using the well-known terephthalic acid (TA) test, consisting of the monitoring of the fluorescence emission of the reaction product 2-hydroxyterephthalic acid (2-HTA). In Fig. 6A the fluorescence spectra recorded after 60 min of irradiation for the investigated photocatalysts are reported. In the absence of a catalyst (blank), no significant fluorescence signal is observed, as expected. In contrast, in the presence of the catalysts a pronounced emission band centered at approximately 425 nm appears, which can be attributed to the formation of 2-HTA and therefore to the generation of hydroxyl radicals.

**Fig. 6:**
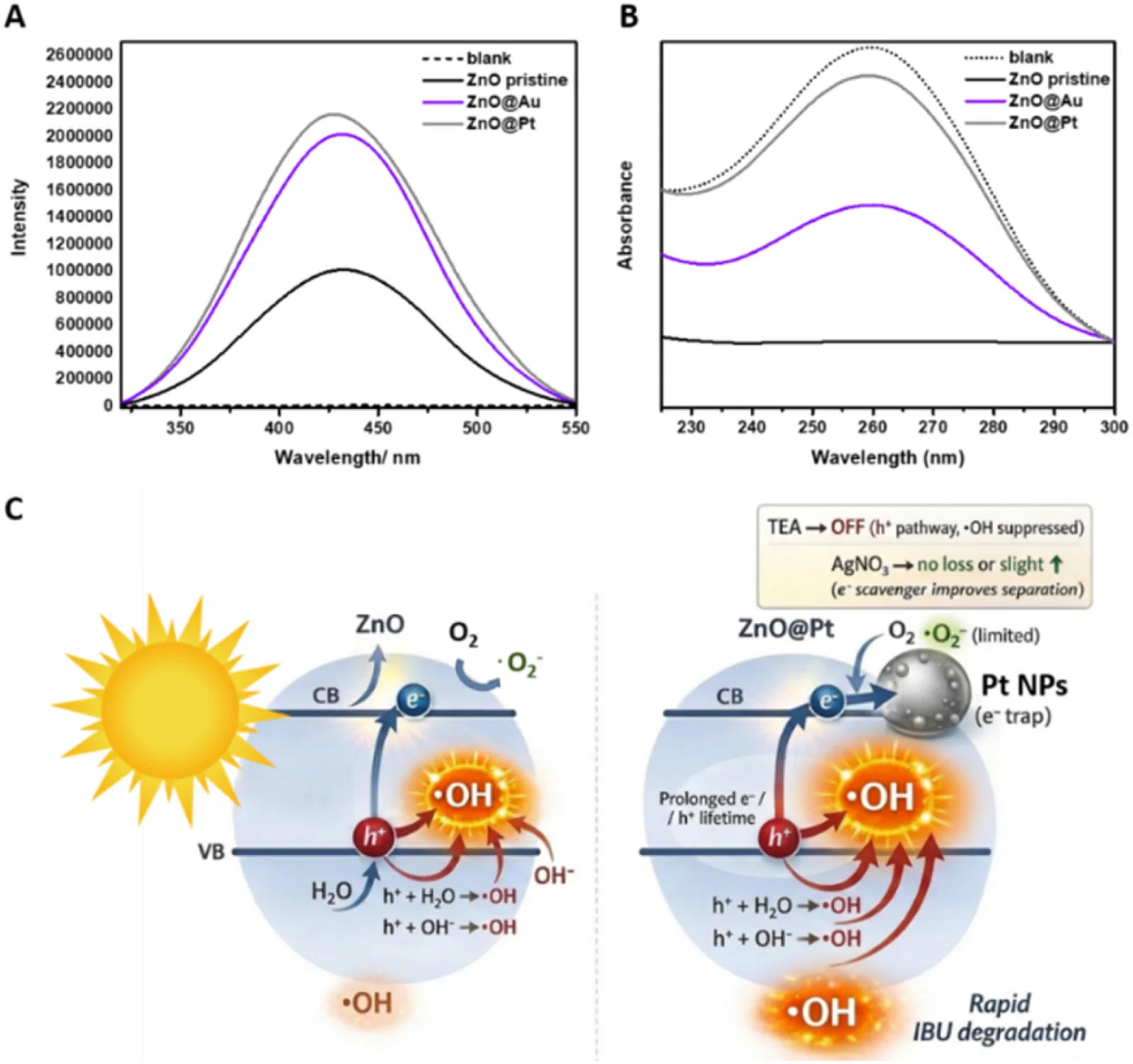
(A) Fluorescence spectra of TA solutions after 60 min of irradiation in the presence of the photocatalysts investigated; (B) UV-vis spectra of NBT solutions recorded after 60 min of irradiation with the same photocatalysts, compared with the non-irradiated blank, used to assess the formation of superoxide-related reducing species; (C) schematic illustration of the proposed photocatalytic degradation mechanism of IBU over ZnO and ZnO@Pt, highlighting charge separation, reactive oxygen species generation and the enhanced – OH-mediated pathway on Pt-decorated ZnO. Scheme reported in panel C was created with the assistance of FigureLabs.

Significant differences in the production of 2-HTA fluorescence intensity are observed for the three materials. In the presence of pristine ZnO, the 2-HTA emission signal remains relatively weak, indicating a limited formation of ˙OH radicals. In contrast, when ZnO@Au is used as the catalyst, the 2-HTA fluorescence intensity is nearly twice that obtained with pristine ZnO, while ZnO@Pt displays the strongest emission signal, indicating a progressively enhanced production of hydroxyl radicals in the presence of noble metal NPs.

This behavior is consistent with the electronic structure analysis discussed in Section 3.1. The formation of metal–semiconductor interfaces promotes interfacial electron transfer from ZnO to the metal NPs, leading to a more efficient separation of photogenerated charge carriers. The suppression of charge recombination increases the lifetime of photogenerated holes, which can subsequently oxidize surface hydroxyl groups or water molecules to generate highly reactive hydroxyl radicals. The particularly strong 2-HTA fluorescence observed when ZnO@Pt is used as the catalyst suggests a more efficient generation of ˙OH radicals, which is in excellent agreement with the photocatalytic degradation experiments and the kinetic analysis reported in Section 3.2. The enhanced formation of reactive oxygen species therefore provides additional evidence for the beneficial role of Pt NPs in promoting photocatalytic oxidation processes.

The formation of superoxide-derived species was investigated by monitoring the UV-vis spectra of NBT after photocatalytic irradiation in the presence of the different catalysts (Fig. 6B). The non-irradiated blank solution, containing NBT at the initial concentration without a photocatalyst, exhibited a pronounced absorption band in the 240–280 nm region, which can be ascribed to the oxidized form of the probe. After 60 min of irradiation in the presence of pristine ZnO, the absorption band related to NBT appears drastically reduced, indicating an extensive consumption/reduction of NBT by photogenerated reducing species, consistently with a high concentration of superoxide species able to react with the probe. A markedly different behavior was observed for the metal-modified samples: in the case of ZnO@Au, the decrease in the NBT band was less pronounced than for pristine ZnO, and this reduction appears even less intense for the ZnO@Pt catalyst. Although all materials are capable of generating reactive reducing species under irradiation, the amount of superoxide detectable by the NBT assay is substantially lower for ZnO@Au and ZnO@Pt than for pristine ZnO.

Overall, ZnO@Pt promotes a more efficient oxidative pathway than pristine ZnO and ZnO@Au under identical irradiation conditions. In ROS detection tests, ZnO@Pt shows the highest efficiency in terephthalate hydroxylation, whereas the NBT assay reveals that superoxide-related reducing species are less prominent than in pristine ZnO. These findings are consistent with an IBU degradation mechanism mainly driven by valence-band holes and ˙OH mediated oxidation rather than by ˙O_2_^−^. At the same time, its photocatalytic activity is more strongly suppressed by the hole scavenger (TEA) and enhanced by the electron scavenger (AgNO_3_), confirming that Pt decoration primary favors the generation and utilization of oxidative species associated with photogenerated holes.

These findings confirm that the photocatalytic degradation of IBU proceeds predominantly through an oxidative pathway driven by holes and hydroxyl radicals, as also supported by the literature.^11,19,81^ On the other hand, superoxide plays only a secondary role. Consistently, the pronounced PL quenching observed for ZnO@Pt (Fig. 2C) indicates a more efficient separation of photogenerated charges and a longer lifetime of valence band holes, which can thus more effectively sustain the oxidative degradation pathway (Fig. 6C).

When moving from ultrapure water to real water matrices, the performance of photocatalytic processes is often negatively affected by background constituents such as inorganic ions and dissolved organic matter.^82^ Species like bicarbonate, chloride and sulfate can compete with the target pollutant for reactive oxygen species and/or act as scavengers of hydroxyl radicals and photogenerated holes, while also competing for adsorption sites on the photocatalyst surface.^81,83–88^ As a result, these matrix-related effects almost invariably lead to lower degradation efficiencies in real water matrices than in ultrapure water, unless the operating conditions or the photocatalyst formulation are specifically optimized to mitigate matrix effects.^82,85^ To assess the robustness of ZnO@Pt under such more demanding conditions, photocatalytic IBU degradation tests were carried out in commercial mineral water (Acqua Frasassi) spiked with IBU at 1 × 10^−4^ M, retaining the same catalyst loading and irradiation conditions reported for the IBU photodegradation in Milli-Q water (Section 2.4.1). Under these more realistic conditions, ZnO@Pt still promotes a marked IBU removal, reaching ≈30% degradation after 30 min, 56% after 60 min and 75% after 90 min of simulated solar irradiation (Fig. S11A).The degradation profile in mineral water is linear in the 30–90 minute interval (Fig. S11B), as confirmed by a linear fit of *A*/*A*_0_*vs.* time (*R*^2^ = 0.99), indicating that the photocatalytic process remains reasonably fast despite the presence of interfering species. Although the degradation efficiency appears reduced if compared with the experiment performed on IBU-spiked ultrapure water samples, the observed removal degrees confirm that ZnO@Pt retains a high photocatalytic efficiency even in a more realistic water composition, underscoring its robustness and practical relevance for IBU abatement.

## 4 Conclusions

Ultrasound-assisted noble-metal decoration of ZnO yields hybrid photocatalysts with markedly enhanced activity for ibuprofen (IBU) removal from water. Au and Pt nanoparticles are rapidly anchored onto pre-formed ZnO microstructures without altering the wurtzite phase or flower-like morphology, and the optical band gap of ZnO remains essentially unchanged, indicating that the semiconductor electronic structure is preserved. Cut-off filter experiments and photoluminescence quenching reveal that both Au and Pt primarily act as efficient sinks for photogenerated electrons rather than as independent light absorbers, thereby improving charge separation at the ZnO/metal interface. Under the selected conditions, ZnO@Pt outperforms ZnO@Au and pristine ZnO by factors of about 2.5 and 10, respectively, and retains significant photocatalytic efficiency in spiked mineral water, highlighting its robustness under more realistic conditions. Overall, noble-metal decoration of ZnO *via* this piezo-assisted route offers a fast, straightforward and environmentally friendly strategy to access ZnO-based hybrid photocatalysts in which the cooperative action of the oxide support and noble-metal nanoparticles substantially improves IBU removal in both model and real aqueous matrices.

## Author contributions

The manuscript was written through contributions of all authors.

## Conflicts of interest

The authors declare no competing interest.

## Supplementary Material

NA-008-D6NA00485G-s001

## Data Availability

The data supporting this article have been included within the manuscript and the supplementary information (SI). Additional data are available from the corresponding author upon reasonable request. Supplementary information is available. See DOI: https://doi.org/10.1039/d6na00485g.
