# Supplementary material for: One minute ultrasound-assisted gold and platinum-decorated ZnO photocatalyst for ibuprofen degradation
Source: Nanoscale Adv. 2026 Sep 2;8(21):6254–67. doi: 10.1039/d6na00485g (PMC13595589; doi:10.1039/d6na00485g)
Supplement: Supplementary file 1 [file NA-008-D6NA00485G-s001.pdf]

Supplementary Information (SI) for Nanoscale Advances.
This journal is © The Royal Society of Chemistry 2026

One minute ultrasound-assisted gold and platinum-decorated ZnO photocatalyst for ibuprofen degradation

Gabriele Manca[a,b], Valentín García-Caballero[c], Ludovico Valli[a,d], Juan José Giner-Casares[c], Manuel Cano Luna[c]*, Simona Bettini[a,d]*, Gabriele Giancane[d,e]

[a] Dipartimento di Scienze e Tecnologie Biologiche e Ambientali, Università del Salento, Via Monteroni, Lecce, I-73100, Italy

[b] Department of Chemistry, Biology and Biotechnology, Centro di Eccellenza per i Materiali Innovativi Nanostrutturati (CEMIN), University of Perugia, via Elce di Sotto 8, Perugia, 06123, Italy

[c] Departamento de Química Física y T. Aplicada, Instituto Químico para la Energía y el Medio Ambiente (IQUEMA), Facultad de Ciencias, Campus de Rabanales, Universidad de Córdoba, Córdoba, Spain

[d] Consorzio Interuniversitario Nazionale per la Scienza e la Tecnologia dei Materiali, INSTM, Via G. Giusti, Firenze, I-50121, Italy

[e] Department of Cultural Heritage, University of Salento, Via D. Birago 64, Lecce, 73100, Italy

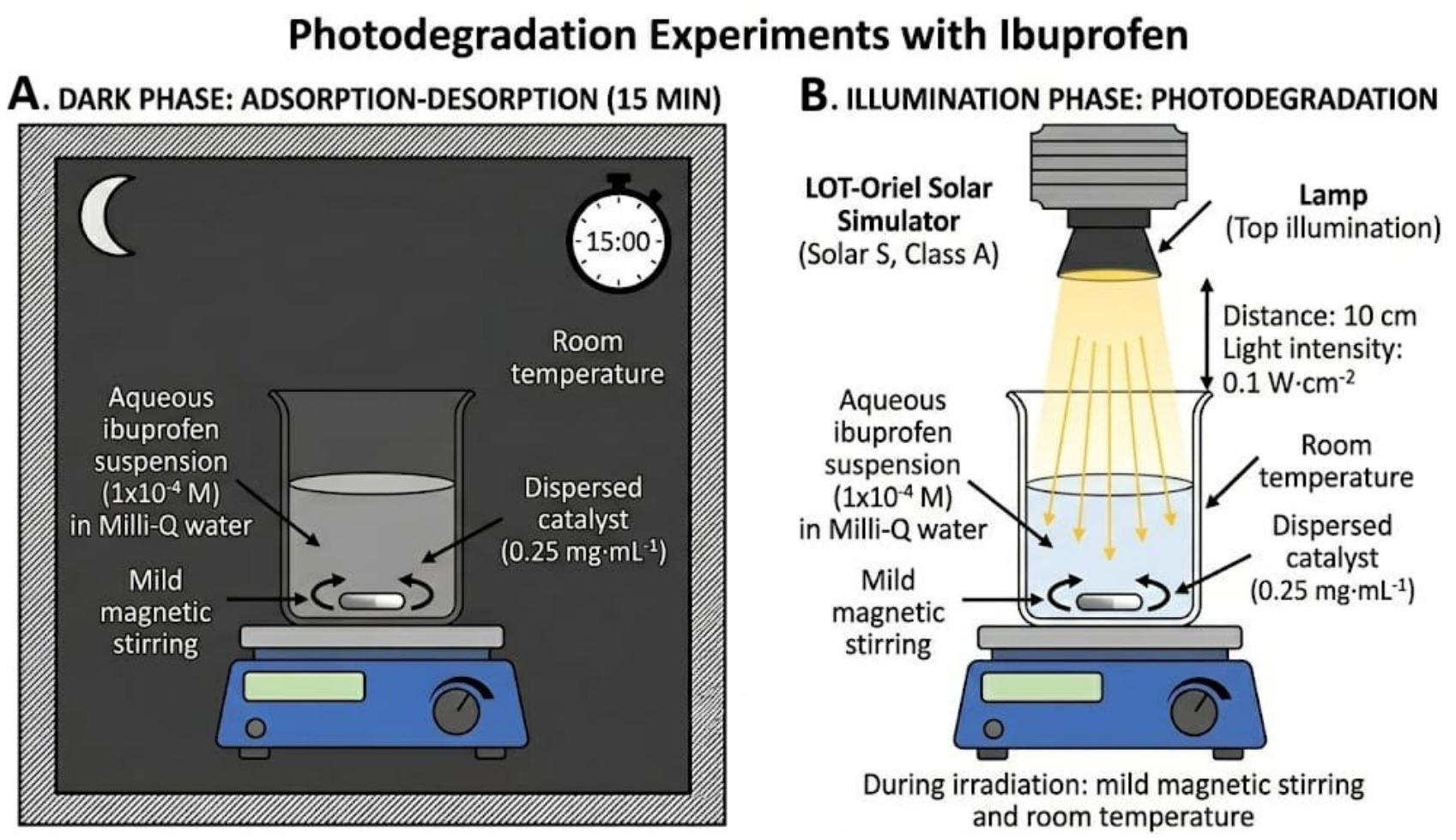

Figure S1 Experimental setup for (A) dark adsorption–desorption equilibrium step and (B) the subsequent ibuprofen photodegradation experiment

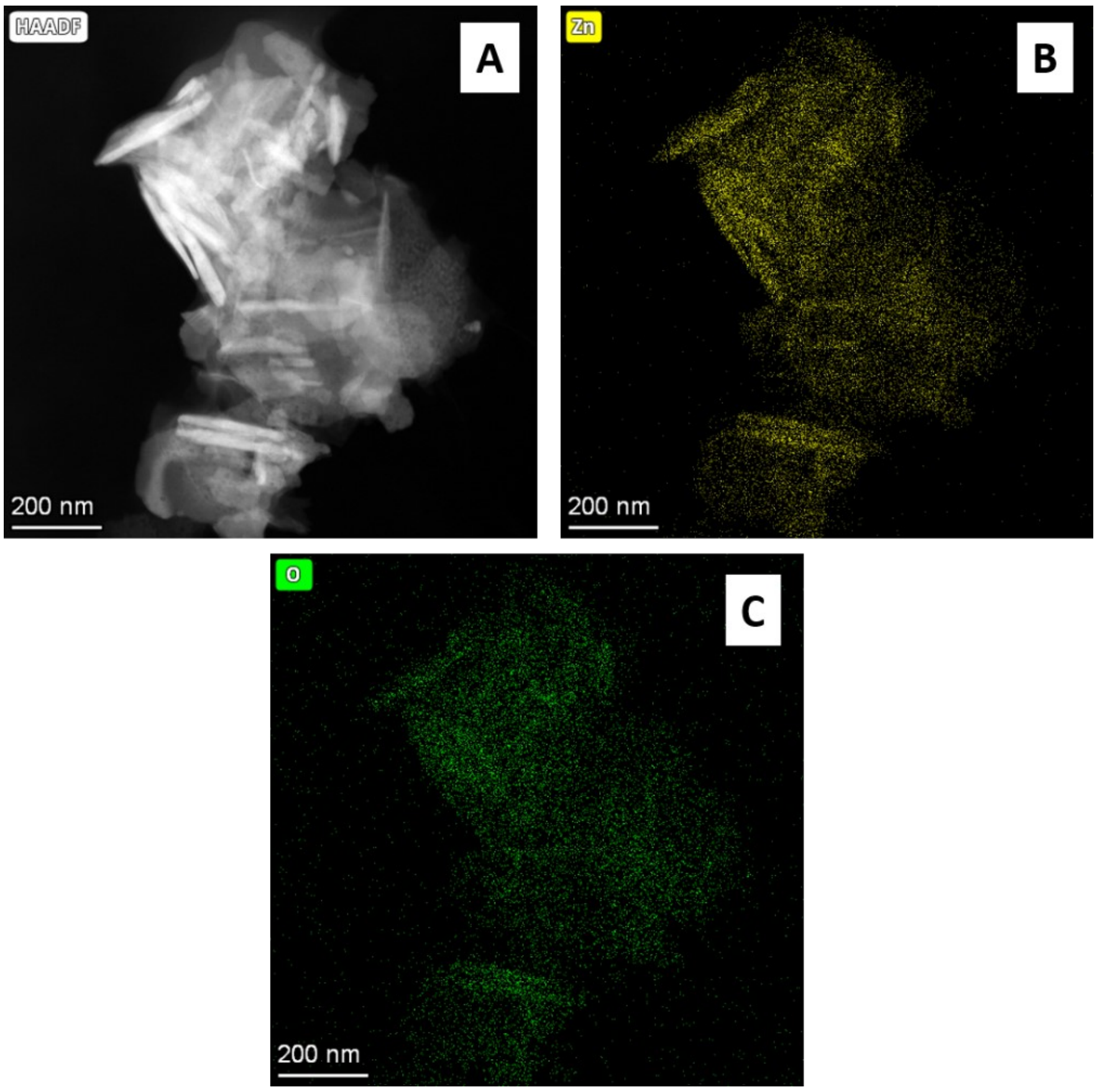

Figure S2 HAADF-STEM of ZnO pristine (panel A) and EDS elemental mapping images (B-C)

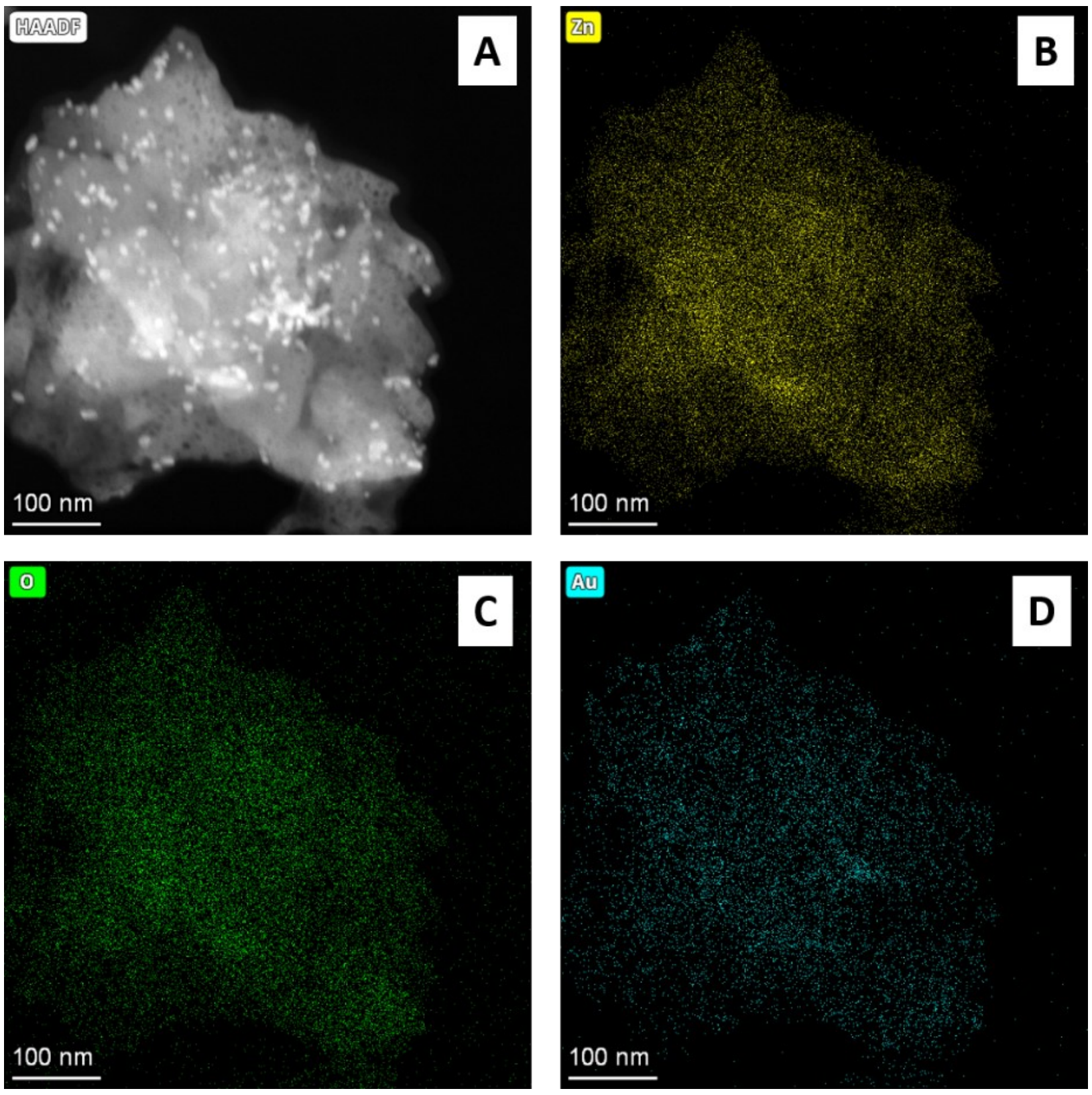

Figure S3 HAADF-STEM of ZnO@Au sample (panel A) and EDS elemental mapping images (B-D)

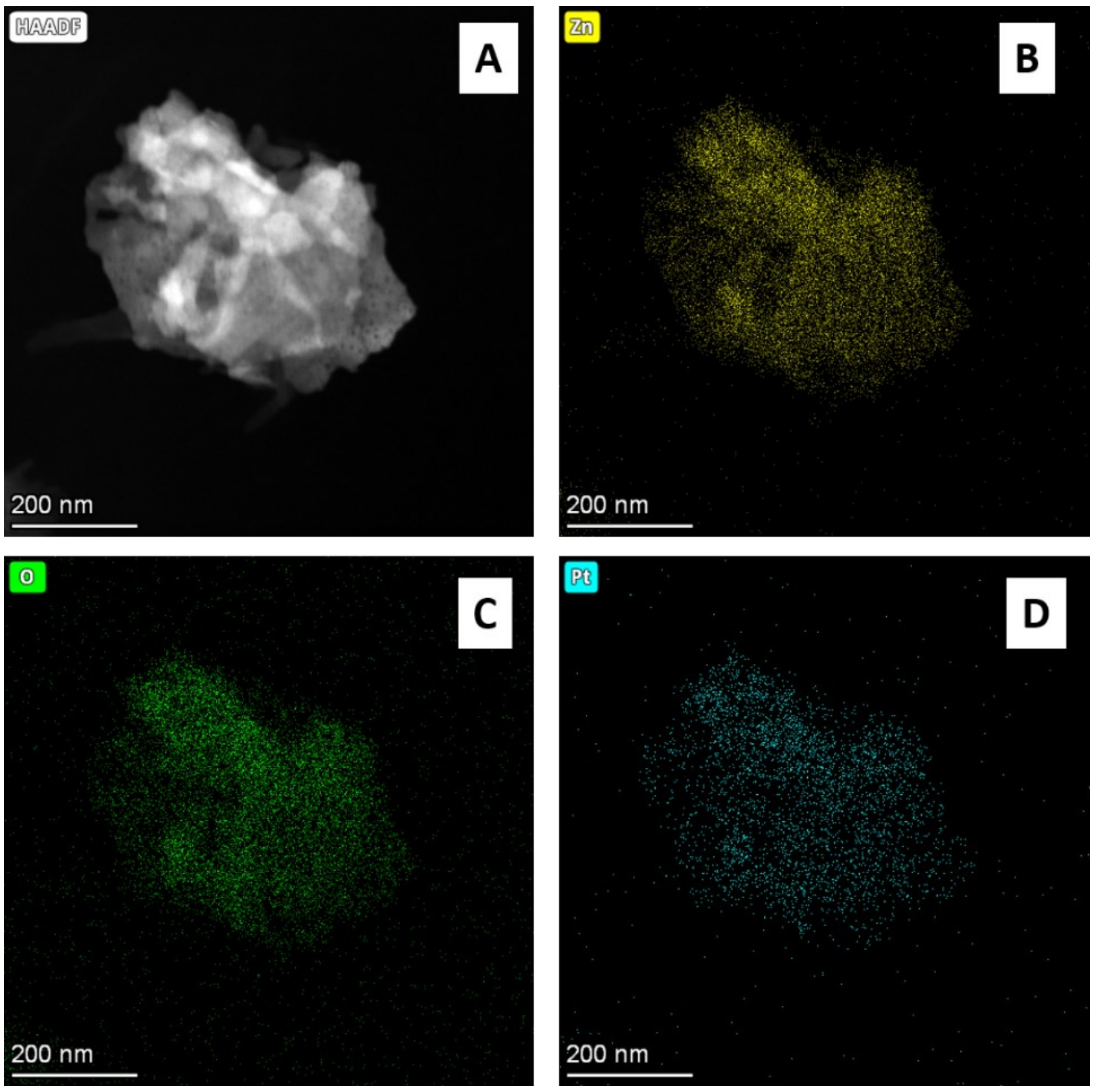

Figure S4 HAADF-STEM of ZnO@Pt sample (panel A) and EDS elemental mapping images (B-D)

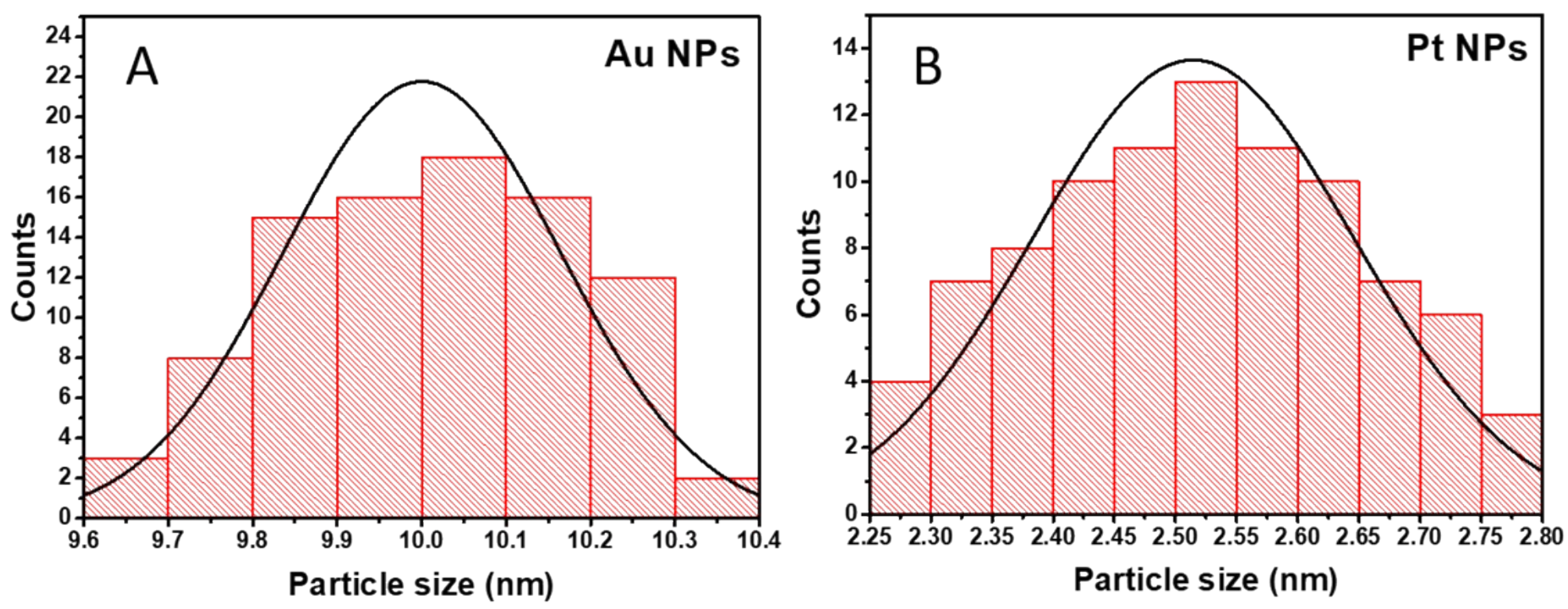

Figure S5 Particle size distributions of Au and Pt nanoparticles (A and B, respectively) in ZnO@Au and ZnO@Pt

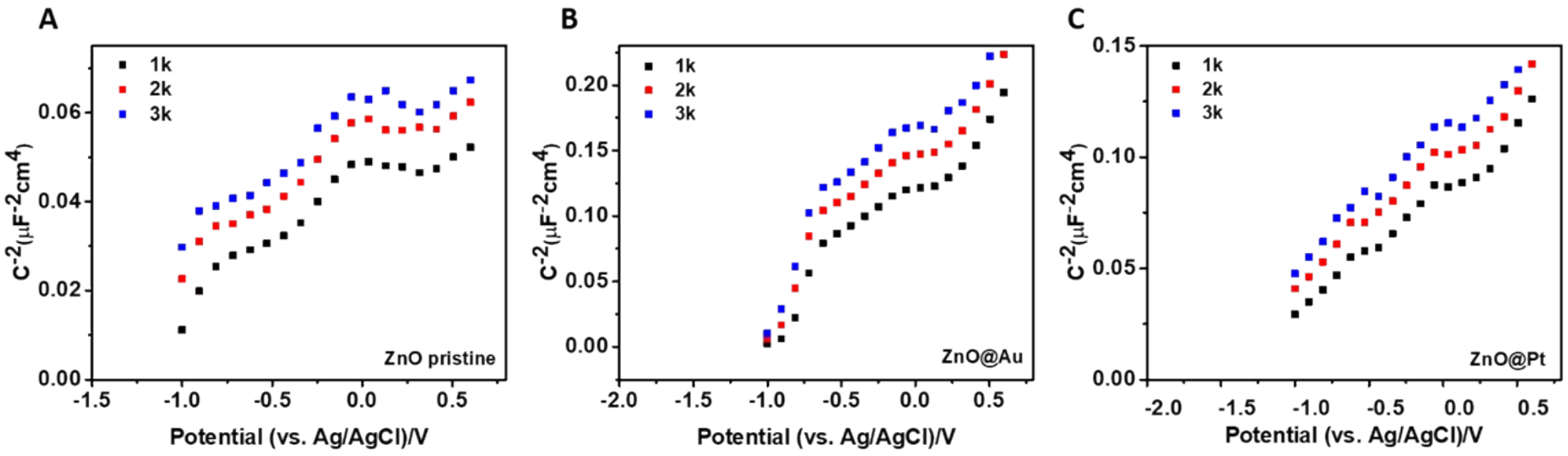

Figure S6 Mott–Schottky plots recorded at different frequencies (1, 2, and 3 kHz) for (A) pristine ZnO, (B) ZnO@Au, and (C) ZnO@Pt electrodes in KOH 0.1 M. The positive slopes observed in all cases confirm the n-type semiconducting character of the ZnO-based materials. The comparable slopes obtained at different frequencies indicate a stable capacitance response and support the reliability of the extracted flat band potentials and donor density values

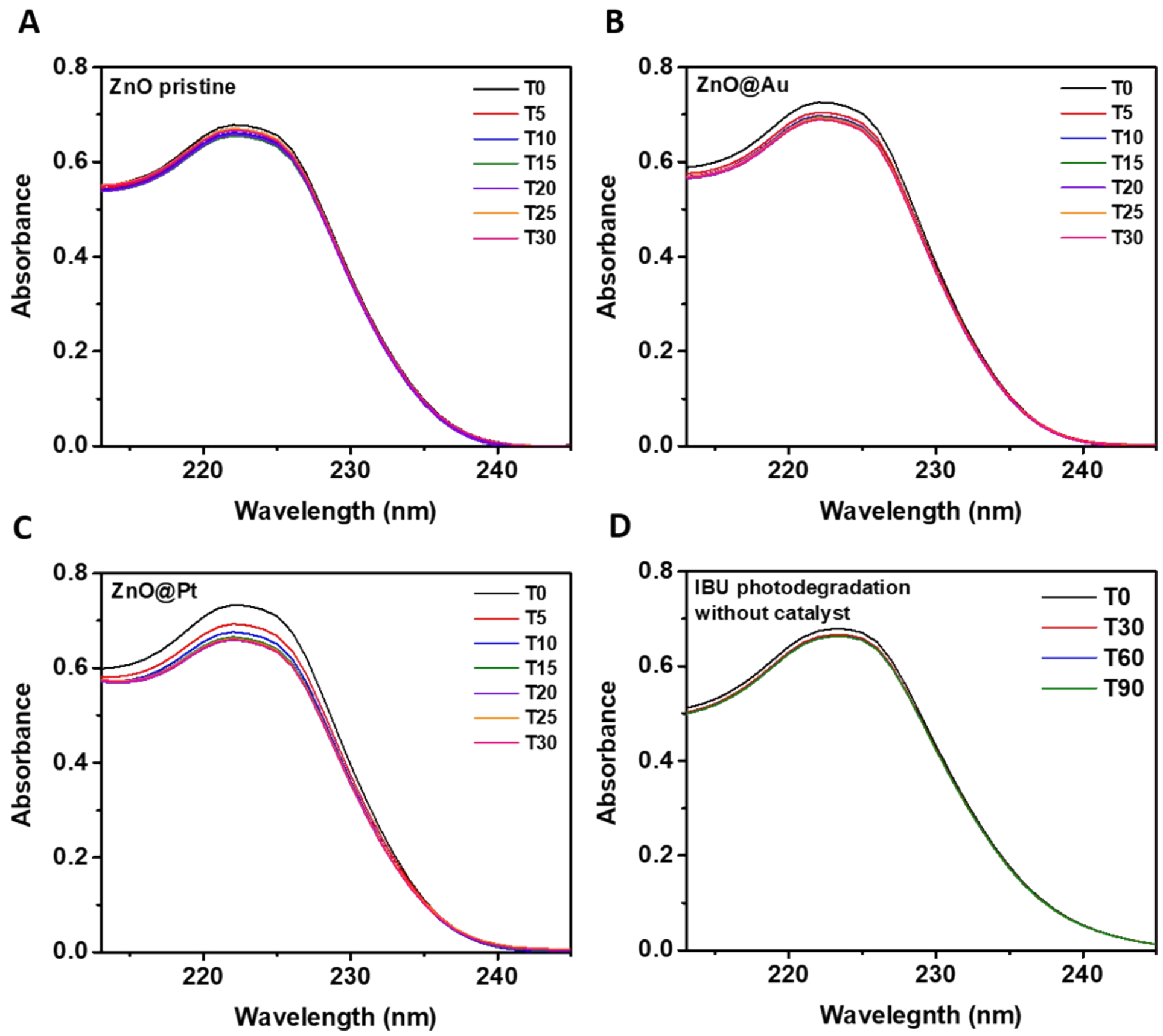

Figure S7 UV–Vis absorption spectra of ibuprofen solutions during dark adsorption tests in the presence of pristine ZnO (A), ZnO@Au (B) and ZnO@Pt (C) The absorbance at ≈225 nm becomes nearly constant after 15 min, confirming that adsorption–desorption equilibrium is reached before photocatalytic irradiation. (D) Photolysis control of ibuprofen under simulated solar irradiation in the absence of any catalyst

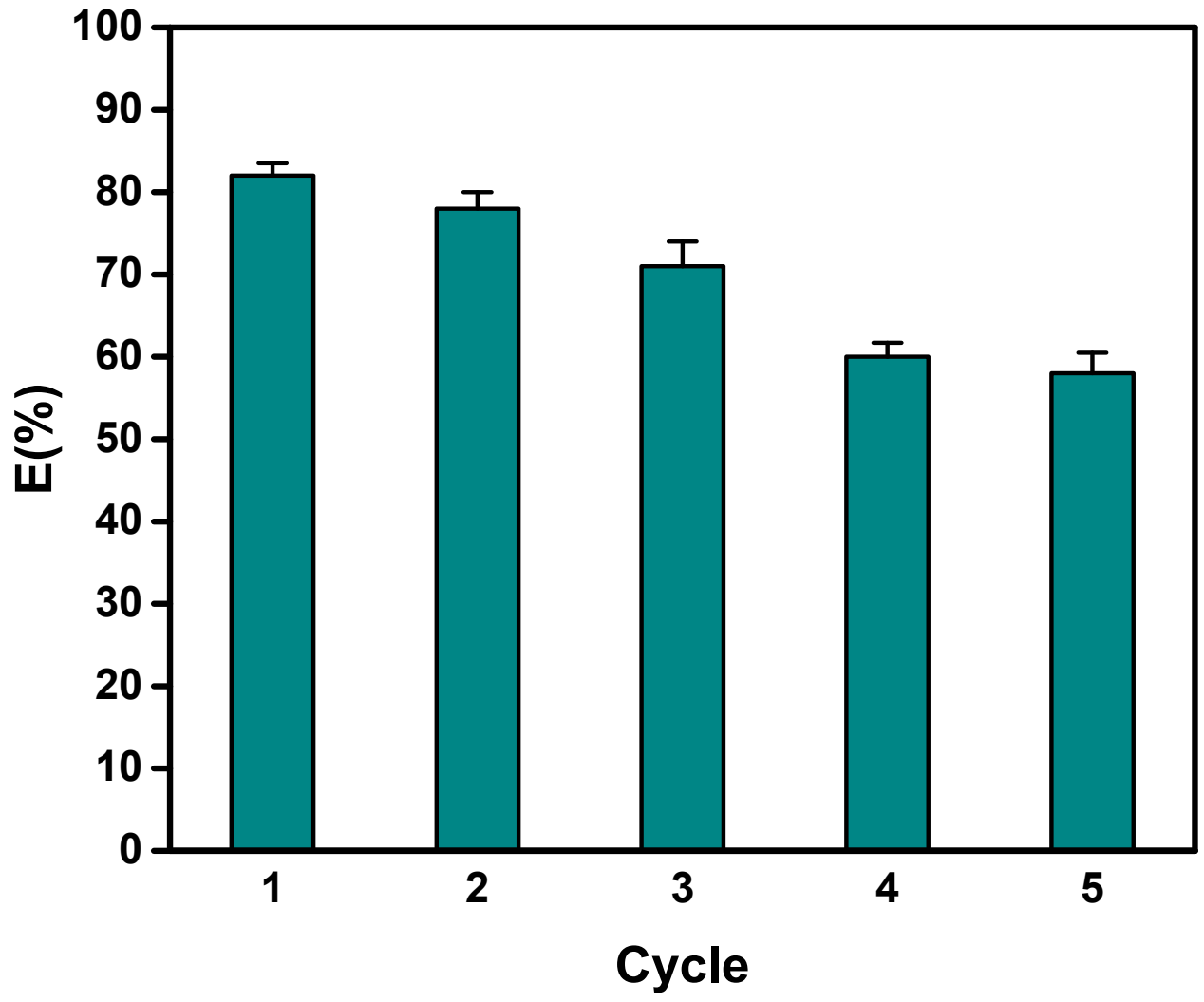

Figure S8 ZnO@Pt catalyst’ s performance over 5 cycles

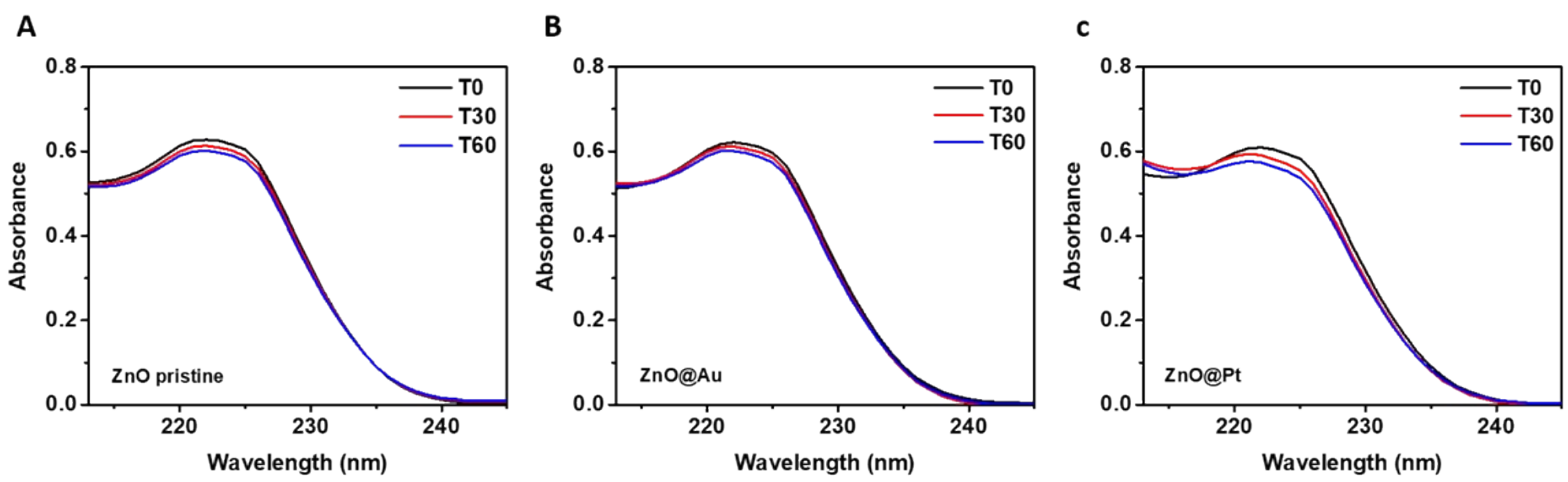

Figure S9 UV–Vis absorption spectra of ibuprofen solutions in the presence of ZnO, ZnO@Au and ZnO@Pt, recorded at 0, 30 and 60 min under irradiation with a 399 nm cut-off filter (λ > 399 nm). Reaction conditions: C_0, IBU = 1×10^{-4} M; catalyst loading = 0.25 mg mL^{-1}

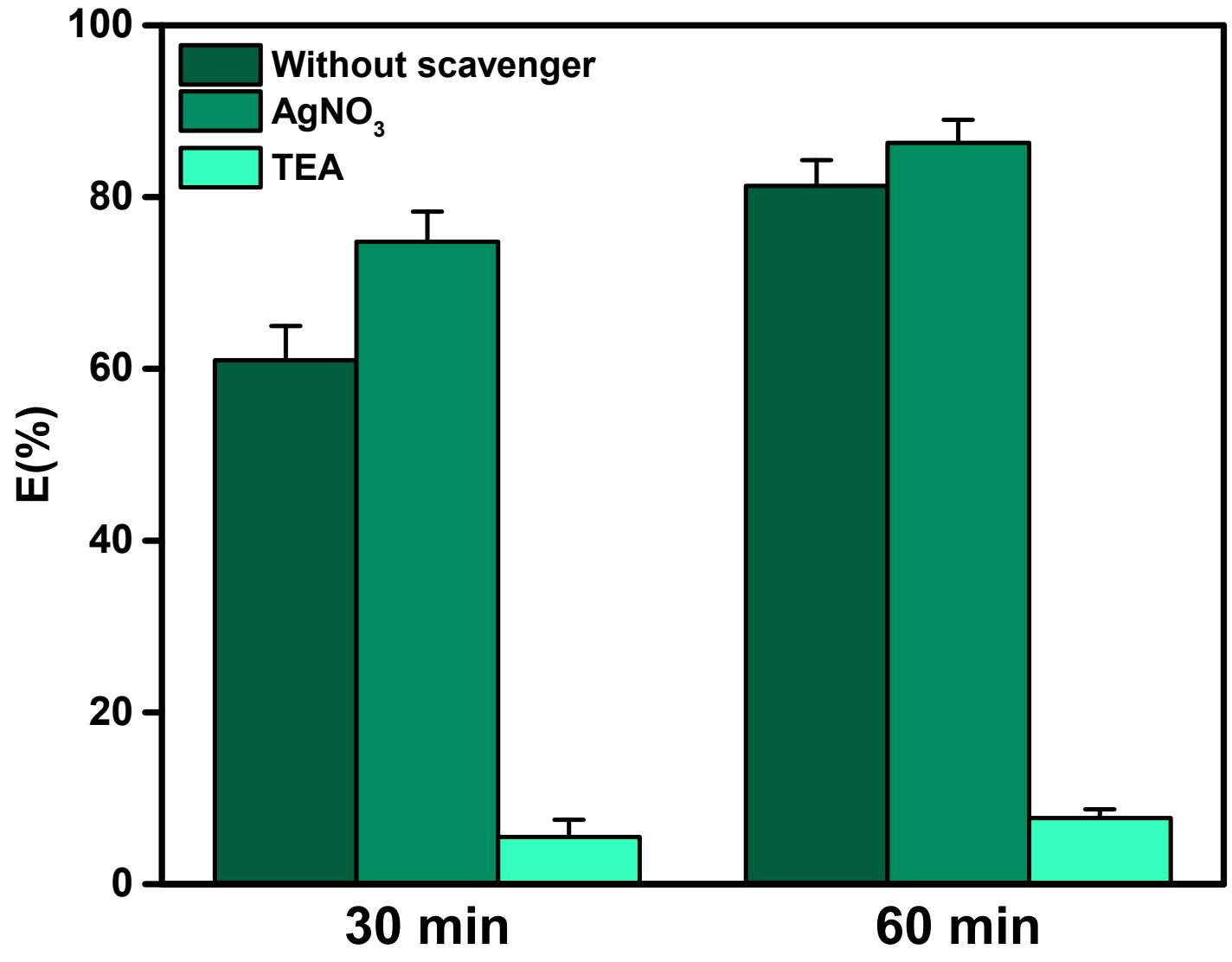

Figure S10 Photocatalytic degradation of ibuprofen after 30 min and 60 min of irradiation in the presence of ZnO@Pt under different scavenging conditions (no scavenger, $AgNO_3$ as electron scavenger, TEA as hole scavenger)

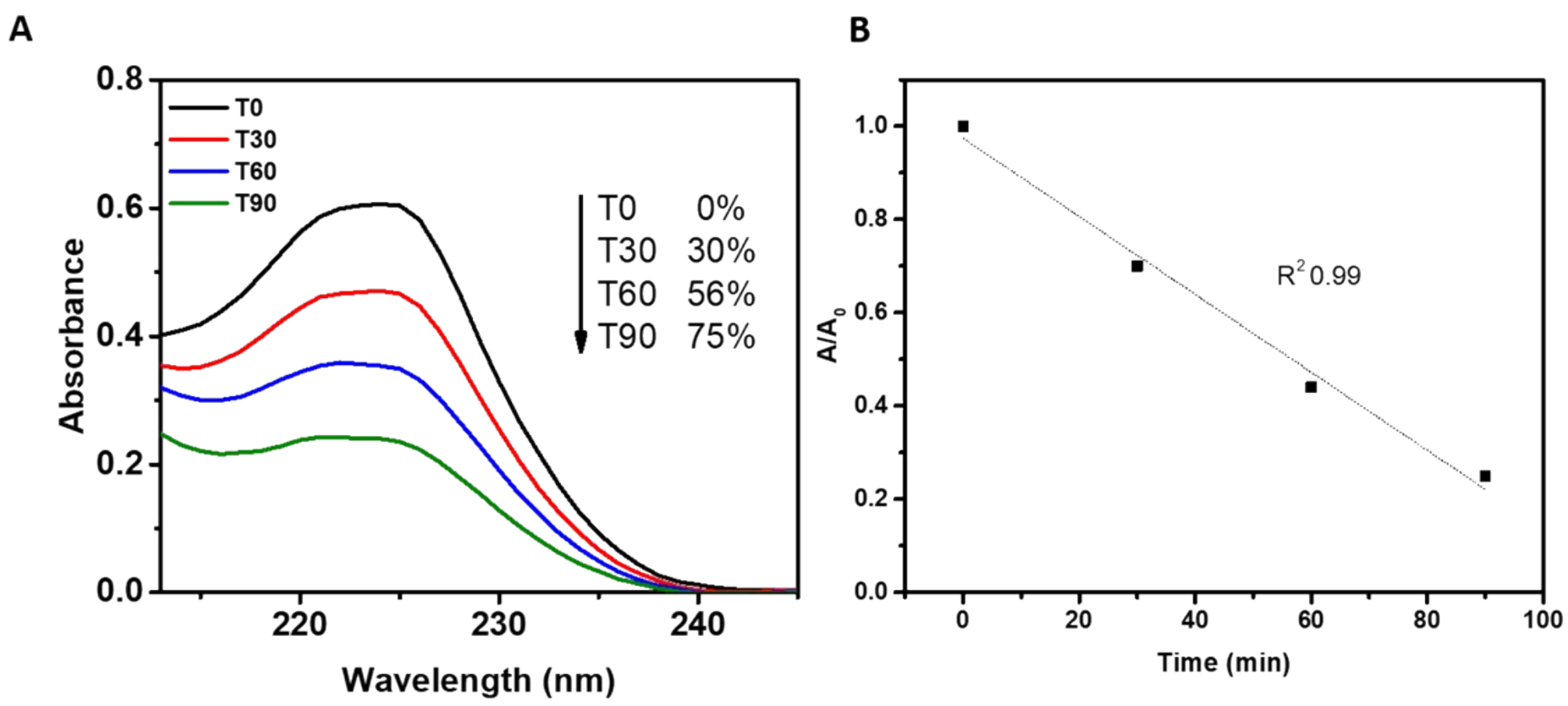

Figure S11 (A) UV–Vis absorption spectra of ibuprofen (C_0, IBU = 1 × 10^{-4} M) in a commercial mineral water in the absence of catalyst and after contact with ZnO@Pt at different stages of the photocatalytic test: after 15 min dark adsorption–desorption equilibrium (T0) and after 30, 60 and 90 min of irradiation (T30, T60, T90). (B) A/A_0 values according to the Beer–Lambert law

Table S1 Main physico-chemical properties of the commercial oligomineral water used in the photocatalytic experiments, as reported on the manufacturer's label.

**Parameter**	**Value (label)**
pH	7.3-7.4
Fixed residue at 180°C (mg L^{-1})	≈330
Conductivity at 20°C (µS cm^{-1})	≈510-520
HCO_3^- (mg L^{-1})	≈310
Ca^{2+}(mg L^{-1})	≈97
Na^+ (mg L^{-1})	≈18
Cl^- (mg L^{-1})	≈20
Mg^{2+} (mg L^{-1})	≈4

Table S2 Summary of the optical and energetic parameters of ZnO pristine and metal-decorated samples

**Sample**	**E_g*(eV)**	**E_{fb} (eV *vs* vacuum)** **	**VB (eV *vs* vacuum)** **	**ϕ metal (eV)*** **	**N_d (10^{20} cm^{-3})********
ZnO pristine	3.19	-4.22	-7.41	4[A]	5.7
ZnO@Au	3.17	-3.77	-6.94	5.1[B]	4
ZnO@Pt	3.16	-3.65	-6.81	5.8[B]	5.9

[A] H. Krysova, V. Mansfeldova, H. Tarabkova, A. Pisarikova, Z. Hubicka and L. Kavan, *J. Solid State Electrochem.*, 2024, **28**, 2531–2546.

[B] G. N. Derry, M. E. Kern and E. H. Worth, *J. Vac. Sci. Technol. Vac. Surf. Films*, 2015, **33**, 060801.

* Optical band gap energies (E_g) were determined from Tauc plots constructed from the absorbance data; **Flat band potentials (E_{fb}) were obtained from Mott–Schottky measurements and converted to the vacuum energy scale. Valence band positions (VB) were calculated as VB = CB − E_g, assuming $E_{fb} \approx$ CB for n-type ZnO; ***Metal work function (Φ) values taken from literature data for polycrystalline bulk surfaces; **** Donor density (N_d) values were estimated from the equation $N_D = \frac{2}{\varepsilon_0\, \varepsilon_r\, e\, m}$ were ε_r is the relative dielectric permittivity (assumed to be 8.3 for ZnO), ε_0 is the vacuum permittivity, *e* is the electron charge and *m* is the Mott-Schottky slope.

Table S3 Photocatalytic degradation efficiency E (%), apparent rate constants (kobs) and corresponding half-life values ($t_{1/2}$) for ibuprofen degradation under simulated solar irradiation

	**ZnO pristine**	**ZnO@Au**	**ZnO@Pt**
**E (%)**	8.9	36.1	90.2
**$t_{½}$ (min)**	770	145	28
**k_{obs} (min)**	9.0 x 10^{-4}	4.78 x 10^{-3}	2.48 x 10^{-2}
